# Interconnected Microphysiological Systems for Quantitative Biology and Pharmacology Studies

**DOI:** 10.1038/s41598-018-22749-0

**Published:** 2018-03-14

**Authors:** Collin D. Edington, Wen Li Kelly Chen, Emily Geishecker, Timothy Kassis, Luis R. Soenksen, Brij M. Bhushan, Duncan Freake, Jared Kirschner, Christian Maass, Nikolaos Tsamandouras, Jorge Valdez, Christi D. Cook, Tom Parent, Stephen Snyder, Jiajie Yu, Emily Suter, Michael Shockley, Jason Velazquez, Jeremy J. Velazquez, Linda Stockdale, Julia P. Papps, Iris Lee, Nicholas Vann, Mario Gamboa, Matthew E. LaBarge, Zhe Zhong, Xin Wang, Laurie A. Boyer, Douglas A. Lauffenburger, Rebecca L. Carrier, Catherine Communal, Steven R. Tannenbaum, Cynthia L. Stokes, David J. Hughes, Gaurav Rohatgi, David L. Trumper, Murat Cirit, Linda G. Griffith

**Affiliations:** 1 Department of Biological Engineering, Massachusetts Institute of Technology, Cambridge, MA USA; 2 Department of Mechanical Engineering, Massachusetts Institute of Technology, Cambridge, MA USA; 3 Center for Gynepathology Research, Massachusetts Institute of Technology, Cambridge, MA USA; 4 Research Laboratory of Electronics, Massachusetts Institute of Technology, Cambridge, MA USA; 5 Continuum LLC, Boston, MA USA; 6 Department of Biology, Massachusetts Institute of Technology, Cambridge, MA USA; 7 Department of Chemical Engineering, Northeastern University, Boston, MA USA; 8 Center for Environmental Health Sciences, Massachusetts Institute of Technology, Cambridge, MA USA; 9 Stokes Consulting, Redwood City, CA USA; 10 CnBio Innovations, Hertfordshire, United Kingdom

## Abstract

Microphysiological systems (MPSs) are *in vitro* models that capture facets of *in vivo* organ function through use of specialized culture microenvironments, including 3D matrices and microperfusion. Here, we report an approach to co-culture multiple different MPSs linked together physiologically on re-useable, open-system microfluidic platforms that are compatible with the quantitative study of a range of compounds, including lipophilic drugs. We describe three different platform designs – “4-way”, “7-way”, and “10-way” – each accommodating a mixing chamber and up to 4, 7, or 10 MPSs. Platforms accommodate multiple different MPS flow configurations, each with internal re-circulation to enhance molecular exchange, and feature on-board pneumatically-driven pumps with independently programmable flow rates to provide precise control over both intra- and inter-MPS flow partitioning and drug distribution. We first developed a 4-MPS system, showing accurate prediction of secreted liver protein distribution and 2-week maintenance of phenotypic markers. We then developed 7-MPS and 10-MPS platforms, demonstrating reliable, robust operation and maintenance of MPS phenotypic function for 3 weeks (7-way) and 4 weeks (10-way) of continuous interaction, as well as PK analysis of diclofenac metabolism. This study illustrates several generalizable design and operational principles for implementing multi-MPS “physiome-on-a-chip” approaches in drug discovery.

## Introduction

The failure of pre-clinical cell culture and animal models to predict drug safety and efficacy in humans results in billions of wasted dollars each year and slows development of treatments for needy patients^1–4^. These gaps have driven an explosion of approaches to capture complex human physiology *in vitro*, merging several parallel threads of science and technology, including pluripotent stem cell (PSC) and organoid biology; design principles and tools for 3D tissue and organ culture; microfluidic and mesofluidic approaches to controlling perfusion flow; and quantitative systems pharmacology models^5^. These efforts are driving development of tools often referred to as “microphysiological systems” (MPSs) or “organs-on-chips” (OOCs), which encompass the subset of approaches employing (i) perfusion flow to improve physiology or workflow even for monolayer cultures of a single cell type, and (ii) 3D cultures comprising multiple different cell types, representing a desirable subset of organ or tissue functions (e.g. metabolism or excretion), with or without flow through the system, as recently reviewed elsewhere^6–14^. The term MPS is used here, rather than “OOC,” to avoid the implication that an entire organ system is recapitulated *in vitro*.

Applications in pre-clinical pharmacology and toxicology, with an emphasis on drug metabolism and safety pharmacology, were arguably the early, highly visible drivers of the field and remain prominent in publications and in commercialization efforts^6,7,11,15–25^. For these applications, design of individual MPSs and interlinked multi-MPS platforms has predominantly focused on capturing prominent physiological functions such as absorption, metabolism, or barrier function represented by one or two cell types. However, at least two factors are driving creation of systems with greater biological (and correspondingly technological) complexity. One is the growing recognition that the governing biological phenomena in preclinical pharmacology and toxicology often involve multiple cell types with complex mechanistic crosstalk within a single MPS. For example, standard hepatocyte-centric *in vitro* liver models fail to capture the deleterious interplay between the immune-targeting drug Tocilizumab and metabolism of small molecule drugs that emerged after Tocilizumab entered the clinic^20^.

A second important factor driving development of more complex systems is that drug failures mostly arise from lack of efficacy, not drug toxicity^3^. This is especially true of cancer and chronic diseases without clear single gene defects, such as diabetes, Alzheimer’s, and arthritis, where multiple organs and tissues may be involved in the pathology. Thus, disease modeling, with the aim of evaluating targets and efficacy, is emerging as a prominent frontier for the field, driving development of complex MPSs with perfusable microvascular networks and other higher-order tissue functions^26–28^.

In tandem, multi-MPS platforms are evolving to support greater complexity of MPS configurations for analysis of multiple modes of organ-organ crosstalk^11,29–36^. These platforms bring with them a host of both conceptual and technical challenges. Conceptually, the first challenge is framing the physiological problem appropriately – the minimal MPS compositions and formats needed to determine clearance of a small molecule drug are rarely the same as those needed for biological therapeutics, for example, and efficacy evaluations are all unique. Second, scaling MPSs (tissue:tissue and tissue:media ratios) to obtain adequate *in vitro*-*in vivo* translation (IVIVT) remains a challenge without a universal solution. The most common scaling approaches are direct miniaturization and allometric scaling^37–40^. However, an alternative scaling approach, rooted mechanistically in the functional aspects of each MPS in an application-based context, may provide more versatility to accommodate the diversity of applications and range of experimental constraints encountered in multi-MPS platform implementation^41^.

Technical challenges in building functional multi-MPS platforms include: (i) creation and maintenance of MPSs that exhibit sufficiently representative and robust physiological function over extended culture periods, typically requiring resource-intensive procurement and preparation of primary cells or pluripotent-stem cells (PSCs) to reach functional maturity in specialized microenvironments; (ii) design and fabrication of platform hardware that can accommodate and sustain the relevant MPSs – including transfer from off-platform to on-platform for MPSs requiring disparate maturation times and complex maturation media – while fluidically linking them together in a manner that is permissive for quantitative analysis of biological phenomena involving drug fate or disease phenomena; (iii) selection of a medium composition compatible with the different MPSs on the platform; (iv) a variety of other practical and translational aspects including flow partitioning, flow rates, physiological sensors, sampling frequency, and sample volume, among others.

**Figure 1:**
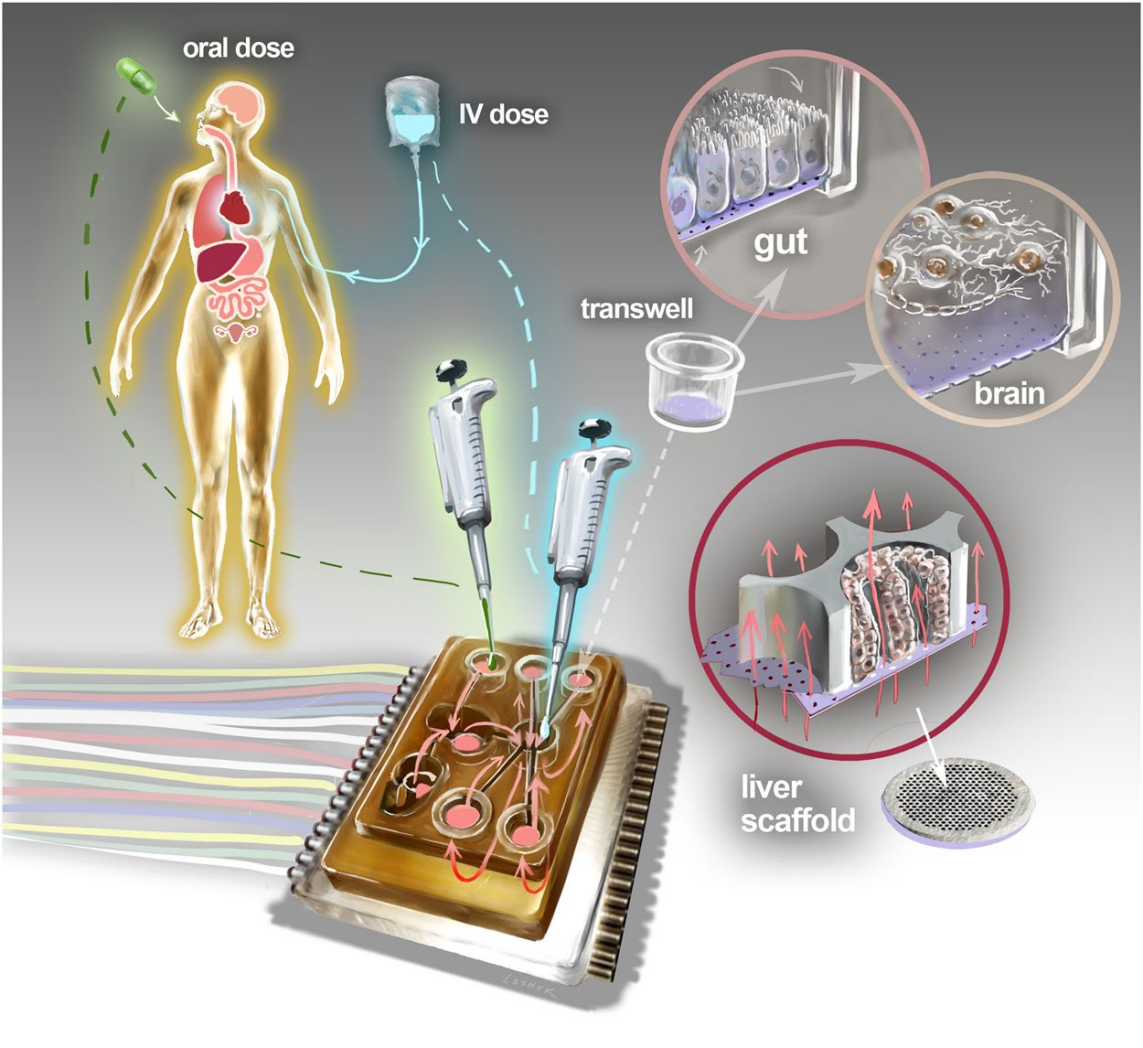
Schematic overview of Physiome-on-a-chip approach. The Physiome-on-a-Chip comprises bioengineered devices that nurture many interconnected 3D MPSs representing specified functional behaviors of each organ of interest, designed to capture essential features of *in vivo* physiology based on quantitative systems models tailored for individual applications such as drug fate or disease modeling. By interconnecting MPSs, dynamic multi-organ signaling can be recreated naturally through cytokine and hormone circulation, cell trafficking, and metabolic byproducts. Multi-MPS systems bridge the complexity gap between traditional *in*-*vitro* cell culture, animal models, and human patient samples, potentially providing better prediction of human responses at lower financial and ethical costs as compared to current methods of drug development. Illustration by Victor O. Leshyk.

Here, we describe the development and implementation of multi-MPS platforms, aka physiome-on-a-chip, supporting 4-way, 7-way, and 10-way MPS interactions for several weeks in the context of these conceptual and technical challenges, illustrated in Fig. 1. We focus on pharmacological testing and interrogation of MPS crosstalk, thus illustrating the use of quantitative systems pharmacology (QSP) modeling for integrating experimental design and interpretation with platform design and operation. QSP was originally defined as a combined experimental and computational approach in translational medicine to elucidate and apply pharmacological concepts for development and use of therapeutics^42^, but has recently been extended to the development of multi-MPS technologies^5,38^. The empirical pharmacokinetic and pharmacodynamic (PKPD) models currently used to characterize relationships between drug kinetics and biological effects in *in vivo* models are not directly translatable to multi-MPS platforms; rather, the diversity of MPS designs requires the use of PKPD models that include more mechanistic details that capture the interrelated physical dynamics (e.g., flow rates in the MPS) and biological dynamics (e.g., drug metabolism and transport, cytokine/growth factor/hormone production and release) in order to select the appropriate experimental conditions, analyze results, and predict human outcomes^30,43^. QSP experimental design takes into account cell numbers and types in each MPS, working media volumes, and flow patterns (flow-through or closed circuit) among different MPS configurations, and involves characterization of the relationships between drug exposure and MPS responses. The flow partitioning between MPSs and total systemic flowrate, together with MPS configuration (i.e., diffusional or other molecular transport barriers within each MPS) are the operational drivers of drug exposure in each MPS^38^.

The platforms developed and characterized in this work (Fig. 1) derive from the open system, multi-well plate format utilized in Liverchip^®^ technology, which we developed for long-term perfused culture of 3D liver-like tissue^44,45^ and recently extended to 2-way (gut-liver) MPS pharmacokinetics^36^ and inflammation^30^ interactions. These new platforms incorporate a high degree-of-freedom (DOF) on-board pumping system with a wide range of flow rates and low drug binding to precisely control intra- and inter-MPS mixing and distribution. This technology enables simultaneous tuning of flow rates between and within different MPSs in a circulation loop, a feature missing in most active and passive fluid-control systems that have been implemented in multi-MPS systems.

Features of the Liverchip^®^ design retained in this multi-MPS platform are an open-system format constructed with materials refractory to adsorption of lipophilic drugs and hormones and sufficient pumping capacity to support modeling of diseases such as cancer metastasis^46^, where relatively large (1 + million cells) tissue mass is required in each MPS. The platforms accommodate a mixing chamber (“mixer”) for the systemic circulation, a liver module that recirculates flow through a scaffold with 3D primary human liver culture, and up to nine Transwell^®^-format MPS modules with recirculation in the basal compartment. The Transwell^®^ MPS format is attractive for barrier epithelial tissues such as gut, lung, and others, as many established and emerging protocols for constructing intact epithelial barriers exhibiting varying degrees of physiological permeability and excretion have been described using off-the-shelf components. They can be constructed off-platform and moved on-platform at appropriate stage of maturity^31,47,48^. Models of the central nervous system (CNS) and other tissues and organs have also been adapted to a Transwell^®^ format amenable for inclusion on this platform^22,49^.

In the first system, we demonstrate two-week sustained functional maintenance of four interacting MPSs (liver/immune, lung, gut/immune, and endometrium). Using stepwise escalation of the flow rates on the 4-way platform we then show that systemic distribution of secreted proteins follows a predictable profile as the system approaches a more rapidly well-mixed state. Next, we demonstrate the first reported co-culture of seven interacting MPSs. This system, consisting of an additional three MPS (brain, heart, and pancreas) to the first four, was able to maintain phenotypically functional cultures for 3 weeks of interaction. Using this 7-way platform, we quantified *in vitro* pharmacokinetics of diclofenac, a non-steroidal anti-inflammatory drug, given as an “oral” dose to the apical side of the gut barrier epithelium. We found strong agreement between the computationally predicted and measured concentrations of the drug and its metabolite in the system.

Finally, a 10-way MPS platform was configured to include kidney, skin, and skeletal muscle MPSs, in addition to the 7 MPSs described above. The 10-way platform was successfully operated in continuous interaction mode to maintain MPS phenotypic functionality for 4 weeks. Notably, successful operation of this platform relied on robust performance of all 26 individual pumps for almost 5 weeks (including pre-interaction set-up), and design features that minimized risk of contamination during handling, including medium changes and acquisition of over ~1200 fluid samples from the MPS compartments and mixing chamber.

Together, these data support the effectiveness of our approach to multi-MPS experiments using integrated pumping hardware and QSP to enable high-content biology studies for clinically translatable applications in preclinical drug discovery and development. The robustness of the approach to long-term co-culture and analysis of multiple interacting MPSs in a reconfigurable arrangement (as demonstrated by the iterative development of 4-way, 7-way, and 10-way designs) is also appealing for non-commercial applications involving complex biological responses. Although multi-MPS platforms have sometimes been called “human-on-a-chip”, we prefer the term “physiome-on-a-chip” to describe integration of multi-MPS hardware with QSP-type modeling. Even with 10 MPSs, our platform falls short of replicating an entire human, and requires QSP modeling for conceptualizing the relevant physiology and interpreting experimental results.

## Results

### Multi-MPS platform design and fabrication

**Figure 2:**
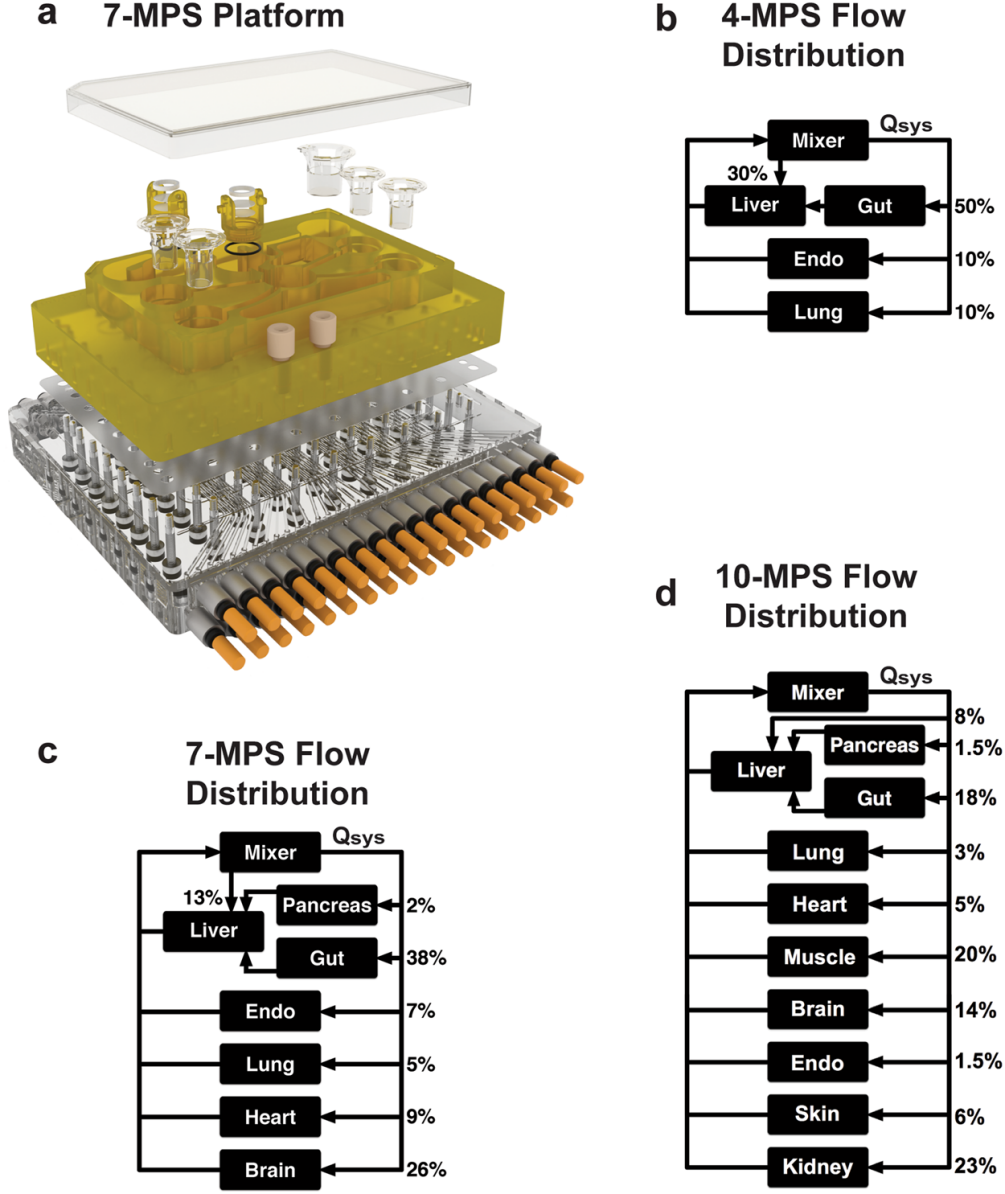
MPS platforms and their flow partitioning. Exploded rendering of the 7-MPS platform (**a**) and corresponding flow partitioning for the 7-way platform (**b**). Rigid plates of polysulfone (yellow) and acrylic (clear) sandwich an elastomeric polyurethane membrane to form a pumping manifold with integrated fluid channels (See Fig. S2 for pump details). Channels interface to the top side of the polysulfone plate (yellow) to deliver fluid to each MPS compartment in a defined manner. Fluid return occurs passively via spillway channels machined into the top plate (See Fig. S2 for details). Flow partitioning mirrors physiological cardiac output for the 4-way (**b**), 7-way (**c**) and 10-way (**d**) platforms. Renderings for the 4-way and 10-way platforms are shown in Fig. S1.

Several desired features of the multi-MPS platform were integrated to define the hardware design. An envisioned application of the multi-MPS platform described here is examination of molecular crosstalk between MPSs via cytokines, growth factors, and other molecular communication modes. This consideration drove an open-system hardware design that allowed relatively straightforward manual sampling of the systemic medium locally within each MPS compartment. An open system also facilitated internal recirculation loops within each MPS to ensure adequate mixing (i.e. in the basal Transwell^®^ compartment or in the flow-through microperfused liver) at internal recirculation rates about 10–100 times greater than flow rates between different MPSs (Figs 2, S1 and S2, Table S1). For epithelial barrier tissues (e.g. gut, endometrium, lung), separately accessible apical and basal compartments, with an intact epithelial barrier that could be characterized periodically by trans-epithelial electrical resistance (TEER), was desired, leading to selection of off-the-shelf Transwell^®^-style configurations. For liver, previous work^20,44,50–52^ suggested that 3D cultures perform better for functional maintenance, with a flow-through microperfusion of the 3D tissue to enhance local exchange of oxygen, nutrients, and drugs independently of systemic circulation rates; hence, the previously developed Liverchip^®^ design was adopted (see below).

Unlike epithelial barrier tissues, there are no standardized MPS formats for the non-barrier MPSs (brain, pancreas, heart, muscle). The use of separate high-capacity recirculation pumps for each MPS enables either a flow-through or a Transwell^®^ format to be assigned at the time of fabrication. For the 7-way platform, a published Transwell^®^-format brain module^22^ was adopted, and a Transwell^®^ module was similarly deployed for the heart and all additional modules on the 10-way platform. A flow-through module (identical to the liver MPS) was adopted for pancreas on the 7-way platform. Although these design considerations resulted in medium volumes and tissue ratios different than those considered elsewhere^37,39,53^, the QSP modeling approaches employed here incorporate the operational characteristics of each MPS in a mechanistic manner to design interaction experiments and data interpretation. Such considerations include the ways in which MPSs carry out physiological functions relevant for the applications, accounting for fluid flow rates and molecular exchange rates, and incorporating intrinsic rates of consumption or production of compounds by relevant cell types in the MPSs.

The multi-MPS fluidic platform used for N-way interactions is a micro-machined bioreactor device with a form factor similar to a microtiter plate, and is built in 3 layers: a top (fluidic) plate, a flexible membrane layer, and a bottom (pneumatic) plate. Expanded renderings of the 4-, 7-, and 10-MPS platforms are shown in Figs 2 and S1. Details of the pumping and fluidics are depicted in Fig. S2. The top layer is machined from a monolithic block of polysulfone (PSF) plastic and includes compartments to accommodate each MPS plus an extra chamber to integrate and mix return flows (mixer), representing systemic circulation. Any remaining space on the top surface can be machined to create a water reservoir to increase local humidity and limit evaporation from the culture medium. Microfluidic channels and pumps are machined into the underside of the top plate and convey fluid from the mixing chamber to each MPS. The individually addressable micro-pumps are fabricated in-line with the fluid channels and are based on the 3-chamber, positive displacement design previously described^45^ and shown in more detail in Fig. S2. Additional pumps below each MPS compartment provide recirculation flow to mix media within the MPS (Fig. S2, Table S1), enhancing nutrient and oxygen transport, and allowing intra-MPS mixing to be controlled independently from the overall systemic recirculation (mixing) rate.

Fluid return and self-leveling of MPS wells are achieved passively by a system of spillway channels on the top surface of the plate that deliver medium back to the mixer (Fig. S2). Spillways eliminate the need for return pumps and level sensors to enforce a balance between influx and efflux while also allowing return flows to cross feed flows in a separate plane. The clear acrylic bottom plate serves as a pneumatic manifold to deliver pressurized air and vacuum locally to each pump, and is bolted to the top plate with screws and spring washers to evenly distribute load. Clamped between the two plates, a thin polyurethane membrane forms the actuation layer and seals the pumps and channels.

The multi-MPS platform operates with multiple pneumatic lines, each of which can be independently set to a pressure or vacuum state (typically +/− 40 kPa), and therefore represents one DOF for fluid control. A single DOF can be used to actuate a valve, while 3 DOF can be used in concert to create a positive displacement pump by actuating two valves and a central pump chamber. Although each individually addressable pump requires 3 DOF, multiple pumps can be run at the same rate by sharing inlets on the pneumatic manifold allowing many pumps to be driven by one triplet of pneumatic lines. Using such a parallel configuration reduces the number of pneumatic switching valves needed in the controller as well as the number of pneumatic tubes entering the incubator. The 4-MPS platform has 9 pumps with 6 separately-programmable flow rates (18 DOF), whereas the 7-MPS platform has 17 pumps with 12 separately-programmable flow rates (36 DOF). The 10 MPS platform also has 12 separately-programmable flow rates (36 DOF), associated with 26 pumps. Greater parallelism in the 10-MPS platform allowed reuse of the same controller as in the 7-MPS platform.

The platforms all accommodate two MPS formats, either flow-through modules or standard Transwell^®^ inserts, and contain a central mixing module downstream of all MPS compartments. The flow-through module format is similar to that previously described, in which on-board pneumatically-actuated microfluidic pumping is used to perfuse culture medium through a scaffold supporting an array of individual 3D liver tissue-like aggregates, such that a near-physiological drop in oxygen tension occurs across the tissue^52^. The scaffold is maintained in a recirculation loop that passes low-oxygen medium across a shallow, open channel, thus providing adequate re-oxygenation before the media returns to the scaffold. Specifications for each MPS configuration and volumes, as well as total system volumes, are in Table S1. Finally, the ergonomics of the platform during handling and sampling were evaluated iteratively throughout the design process to ensure ease of use and to minimize risk of contamination (see Supplemental Video 1).

### Platform Fluidic Performance

To enable effective mixing on the platform and predictable molecular biodistribution among MPSs, it is critical to ensure parity between the intended and actual flow rates as well as high reliability of the individual pumps, as failure of a single pump compromises the entire platform function. Initial characterization of the hardware included direct measurements of pump rates using a capillary tool with a known diameter. Measurements of the height change per unit time give the flow rate. When targeting a flow rate of 1 µL/s (2 Hz), flow rates in thirteen 4-MPS platforms (n = 9 pumps per platform) averaged 0.92 µL/s ± 0.12 µL/s (Fig. S3a). Flow rates in ten 7-MPS platforms (n = 17 pumps per platform) averaged 1.12 ± 0.10 µL/s (Fig. S3b). These deviations are likely attributable to slight machining differences in the depth of the pump chamber. We found that software calibration factors, calculated from an initial measurement, can correct the pump rates to within ±5% of the target flow rates (0.99 ± 0.056 µL/s, Fig. S3c). In practice, we found this low margin of error allows for reliable and deterministic operation, and hence accurate data interpretation. Platforms were stress tested by running all pumps at 2 Hz continuously for ~6 weeks (7.2 million cycles). Final flow rates were found to be within 10% of initial values and no membrane damage was found upon visual inspection. Flow rates for the 10 pairs of plates comprising the 10-MPS systems were found to perform in a similar manner to the 4- and 7-MPS systems (Supplemental Fig. S3d).

### Maintenance of long-term functionality of interconnected MPS

**Figure 3:**
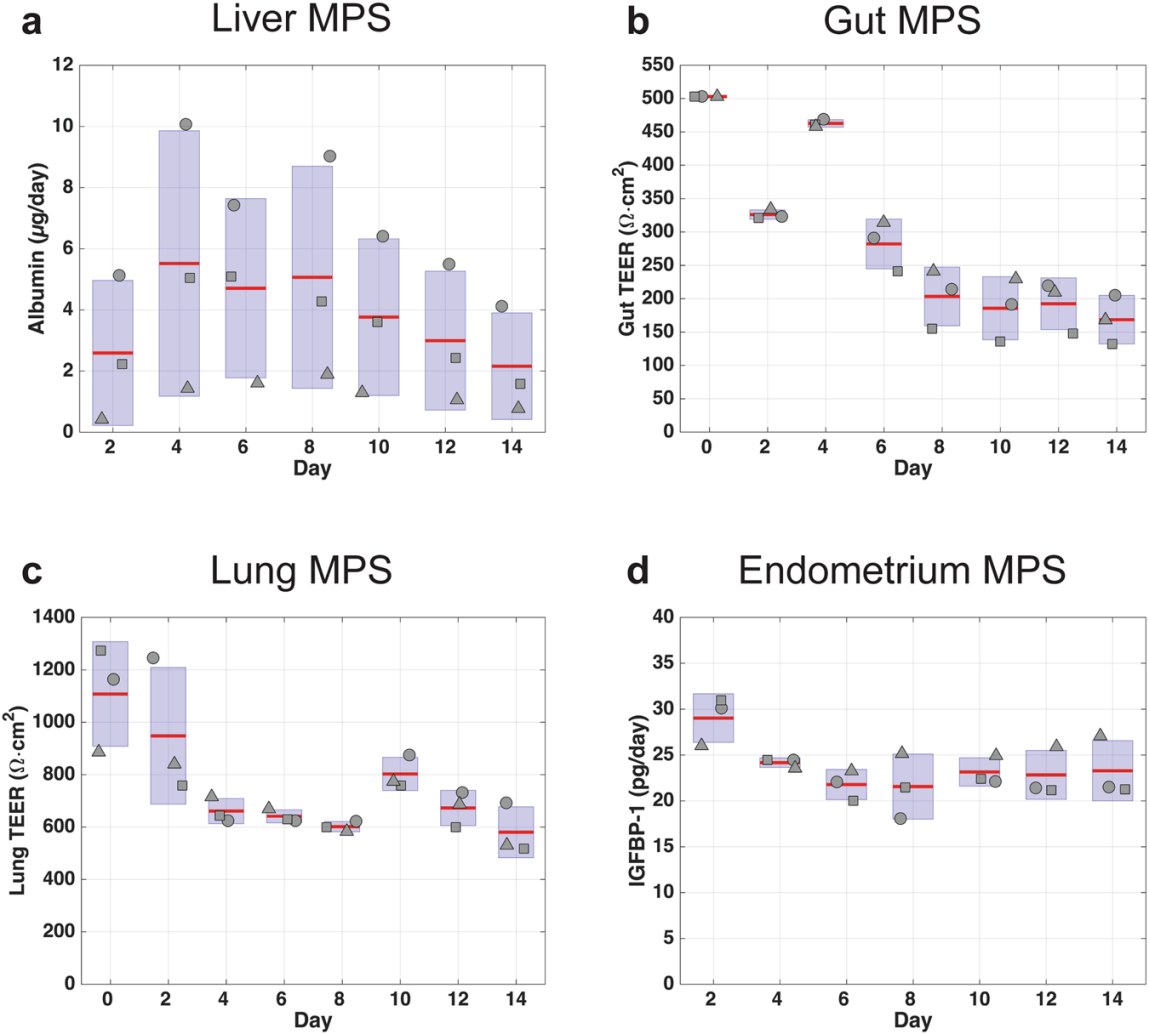
Assessment of MPS functionality in 4-MPS platform. Metrics of tissue function measured during a 2-week co-culture of 4 different MPSs. MPS representing liver, gut, lung, and endometrium were linked using the platform and flow scheme and partitioning shown in Fig. 2. Samples collected from each platform compartment were used to measure protein and metabolite concentrations, which were in turn used to calculate production rates via computational PBPK models. Albumin secretion rates were used as an indicator of liver function (**a**). Barrier functions of gut (**b**) and lung (**c**) MPSs were assessed with TEER measurements. Endometrium MPS functionality was characterized with IGFBP-1 secretion rate to its apical medium (**d**). Compartment and system volumes are in Table S1. The systemic exchange flow rate Q_sys_ was 5 mL/day.

**Figure 4:**
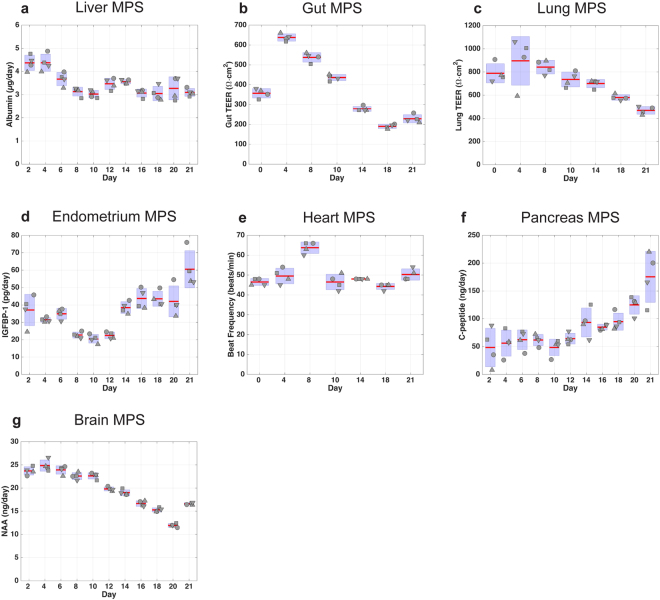
Assessment of MPS functionality in 7-MPS platform. Metrics of tissue function measured for 3-week co-culture of 7 different MPS. MPS representing liver, gut, lung, endometrium, heart, pancreas, and brain were interconnected using the platform, and flow scheme and partitioning shown in Fig. 2 with system and individual compartment volumes indicated in Table S1. Samples collected from each platform compartment were used to measure protein and metabolite concentrations, which were in turn used to calculate production rates via computational PBPK models. Albumin secretion rates were used as an indicator of liver function (**a**). Barrier functions of gut (**b**) and lung (**c**) MPSs were assessed with TEER measurements. Endometrium MPS functionality was characterized with IGFBP-1 secretion rate (**d**) to its apical medium. Heart MPS function was evaluated with beat frequency (**e**). C-peptide production rates (**f**) represented pancreas function. N-acetyl aspartate (NAA) concentrations (**g**) in the apical brain MPS indicated brain MPS functionality. The systemic exchange flow rate Q_sys_ was 10 mL/day.

**Figure 5:**
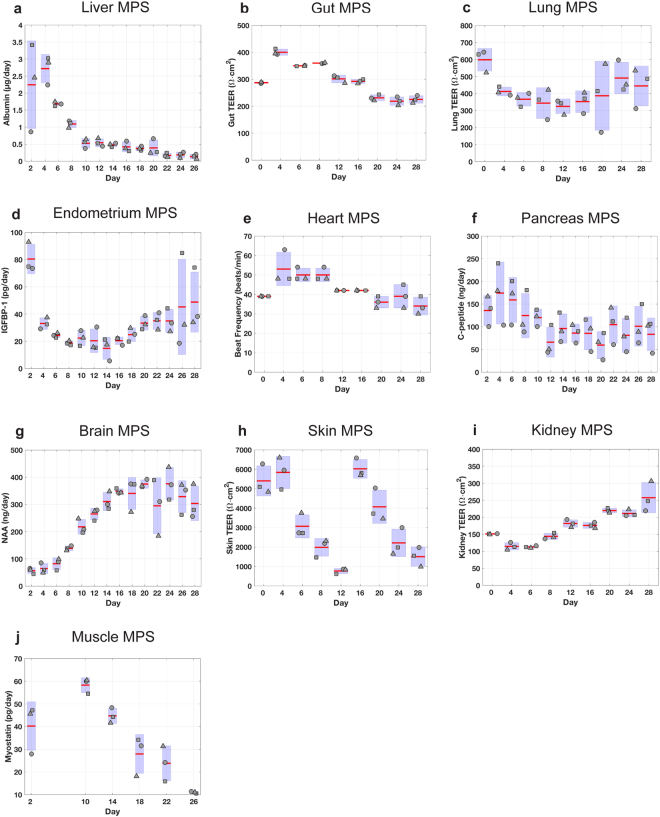
Assessment of MPS functionality in 10-MPS platform. Metrics of tissue function measured for 4-week co-culture of 10 different MPS. MPS representing liver, gut, lung, endometrium, heart, pancreas, brain, skin, kidney, and skeletal muscle were interconnected using the platform, and flow scheme and partitioning shown in Fig. 2 with system and individual compartment volumes indicated in Table S1. Samples collected from each platform compartment were used to measure protein and metabolite concentrations, which were in turn used to calculate production rates via computational PBPK models. Albumin secretion rates were used as an indicator of liver function (**a**). Barrier functions of gut (**b**) and lung (**c**) MPSs were assessed with TEER measurements. Endometrium MPS functionality was characterized with IGFBP-1 secretion rate (**d**) to its apical medium. Heart MPS function was evaluated with beat frequency (**e**). C-peptide production rates (**f**) represented pancreas function. N-acetyl aspartate (NAA) concentrations (**g**) in the apical brain MPS indicated brain MPS functionality. Barrier functions of skin (**h**) and kidney (**i**) MPSs were assessed with TEER measurements. Myostatin secretion was used as an indicator of skeletal muscle function (**j**). The system exchange flow rate Q_sys_ was 20 mL/day.

Prior to assessing responses to interventions, the functional behavior of MPSs in interaction was characterized over time periods of up to 2 weeks (4-way platform), 3 weeks (7-way), and 4 weeks (10-way). The 4-MPS platform studies included liver, gut, lung, and endometrium MPSs, whereas 7-MPS experiments added models for brain, heart, and pancreas MPSs. The 10-way included an additional three systems: skin, kidney, and skeletal muscle MPSs. With the exception of the liver, which was established on platform 3 days before the start of the interaction experiments, all MPSs were matured off-platform and transferred on-platform on the first day of interaction. Cells used in the various MPSs derived from primary sources (liver, lung, pancreas, skin, skeletal muscle, kidney, immune cells in gut and liver), PSCs (heart, brain) and cell lines (endometrium, gut) with different medium needs, and thus no common medium was defined *a priori*. Each MPS was established on the platform in its respective maintenance culture medium at the start of the interaction experiment. During most multi-MPS studies, a systemic interaction flow rate (Q_sys_) of 5 mL/day, 10 mL/day, or 20 mL/day (rates that exchange 60%, 80% and 110% of total system volume per day) for 4-, 7- and 10-MPS studies, respectively, was partitioned to each MPS from the mixer based on the relative percentages of cardiac output to each tissue type in humans (as shown in Fig. 2). Intra-MPS recirculation rates listed in Table S1 were used to enhance mixing; these numbers can be easily modified in the control software for different scaling strategies and MPS modules. Complete media changes were conducted every 48 hours (replacing each MPS’s particular maintenance medium), during which samples were taken to measure secreted biomarker concentration from each MPS, as well as TEER and beat frequency where applicable. Continuous functionality metrics from 4-MPS, 7-MPS and 10-MPS studies are shown in Figs 3, 4 and 5, respectively. In parallel, the functionality of each type of MPS maintained in isolated culture was monitored (Figs S4–S6).

In the 4-MPS interaction study (2-week duration) albumin secretion showed a transient increase in the first few days, followed by a gradual return to initial levels (Fig. 3). Barrier integrity of the gut/immune and lung MPSs was quantified with TEER. TEER values from the gut/immune MPS fluctuated in the early days of interaction studies before settling into a 150–250 Ω · cm^2^ range for the remainder of the experiment. Lung MPS TEER values followed a similar trend of high TEER during the first few days, but eventually established stable values in the 600–800 Ω · cm^2^ range. Endometrium MPS functionality, evaluated by secretion of insulin-like growth factor-binding protein 1 (IGFBP-1), remained around 20–30 pg/day throughout the study. Similar trends for each phenotypic metric were observed in the isolation studies, except IGFBP-1 secretion rate, which was lower in the isolated endometrium MPS (4–12 pg/day) than during interaction studies (20–30 pg/day).

Next, biological compatibility of 7 MPSs (gut/immune, liver/immune, lung, endometrium, heart, brain and pancreas) was assessed in interaction over a 3-week period (Fig. 4). Again, each MPS was differentiated or matured in isolation prior to the interaction study and operated with flow partitioning as shown in Fig. 2. Similar to 4-MPS results, we observed transients in albumin secretion kinetics (with stabilization after a week in this case), sustained gut and lung TEER values, and IGFBP-1 secretion. The beat frequency associated with cardiac muscle cells was used as a functional metric for the heart MPS. We observed that beat frequency was robustly maintained between 45–50 beats/min except for a transient increase to 60–66 beats/min on day 8. N-acetyl aspartate (NAA) and c-peptide release profiles, metabolic indicators of neuron and pancreatic beta cell activity, respectively, revealed that both the brain MPS and the pancreas MPS were also functional for 3 weeks of culture. Comparison of the interaction results with the isolation results showed no negative effect of interaction on the MPS functionality. On the contrary, we observed enhanced NAA (brain) and c-peptide (pancreas) production during the interaction. Due to a dramatic reduction in pancreas MPS functionality in the “isolation” study arm, islets were replaced at day 12 for both interaction and in isolation study arms.

Lastly, 10 MPSs (gut/immune, liver/immune, lung, endometrium, heart, brain, pancreas, kidney, skeletal muscle and skin) were interconnected and the functionality of each MPS was assessed over 4 weeks (Fig. 5). As described above, each MPS was differentiated or matured in isolation prior to the interaction experiment. Similar to our 4- and 7-MPS interaction studies, albumin secretion kinetics was transient. Gut and lung MPS TEER values and trends, IGFBP-1 secretion by the endometrium MPS, and heart beat rate were comparable among interaction studies. In this particular study, unlike the 7-MPS interaction study, the brain MPS comprised iPSC-derived neurons. NAA secretion profile was again sustained and enhanced compared to the isolation study. The pancreas MPS was modified for this study, and 25 islets per MPS were added in a Transwell^®^ format. The observed c-peptide production kinetics were comparable with results from the 7-MPS platform. The barrier function of the kidney MPS, as monitored by TEER, was maintained throughout the experiment, although the TEER values from the interaction study were 3-fold lower than those from isolation studies. Muscle MPS functionality was assessed by myostatin secretion, a myokine released by myocytes, and was well maintained up to day 22, after which secretion decreased during the later stages of the interaction. Barrier function of the skin MPS was transient for both interaction and isolation studies, which suggested the drop in TEER was not due to interaction effects. Thus, the skin MPS was replaced at day 14 with a new skin MPS from a new donor. Similarly, a decline in dendritic cell number, as expected based on their natural half-life^54^, led us to reseed those cells onto the gut MPS on day 19 as described in Methods.

In summary, these findings demonstrated that long-term MPS viability and functionality were maintained in the 4-, 7-, and 10-MPS platforms for applications requiring extended culture periods of up to four weeks.

### Endogenous and exogenous molecular distribution in the multi-MPS platforms

Deterministic operation of multi-MPS platforms via high DOF pumping schemes facilitates *in vitro* data interpretation and translation to *in vivo* outcomes. After establishing that the platforms were mechanically reliable and could support multi-MPS interactions, we investigated the molecular distribution and quantitative measurement of a drug and its metabolite using an integrative approach of quantitative molecular characterization and QSP models.

**Figure 6:**
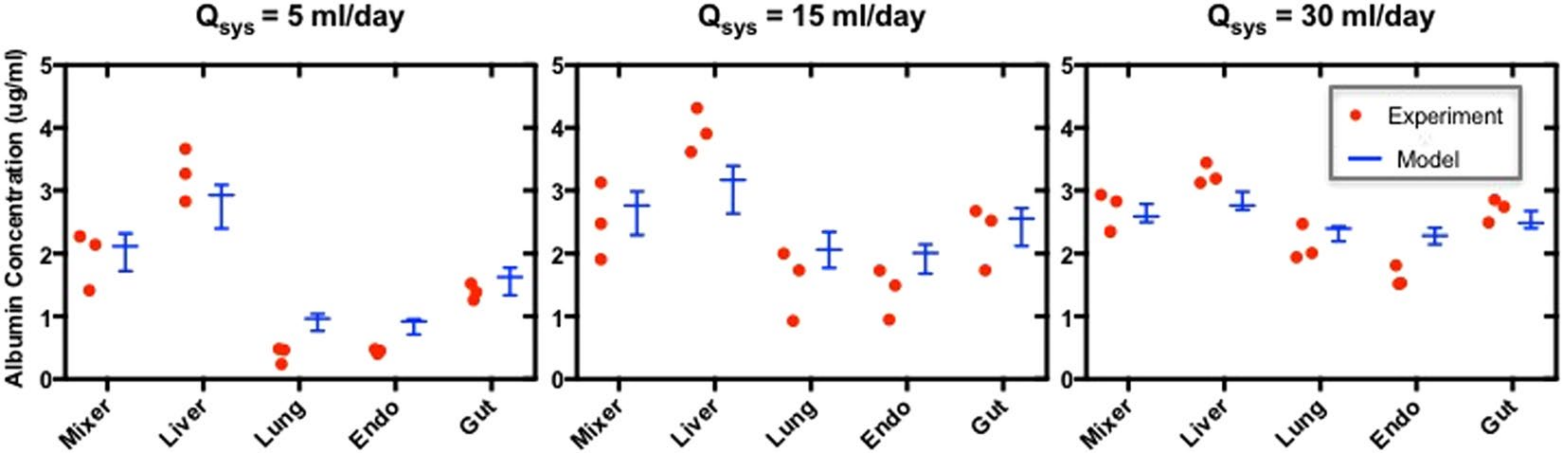
Escalation of systemic flow rate in 4-MPS platform enhances molecular exchange between MPSs without apparent alteration of MPS functionality. Measurements of the concentration of human serum albumin (produced by the liver MPS; red dots) and QSP model fits to the experimental data (Whisker plot indicating median, min-max values) for the mixer and for each individual MPS (liver, lung, endometrium, and gut) for baseline systemic flow rate Q_sys_ = 5 ml/day (left panel) and increased systemic flow rates of Q_sys_ = 15 ml/day (middle panel) and Q_sys_ = 30 ml/day (right panel). The total production rate of albumin is not affected by the flow escalation.

In the 4-MPS platform, the effect of systemic flowrate (Q_sys_) on the distribution kinetics of endogenously-produced albumin was characterized by collecting samples from each compartment every 48 hours during the medium change and analyzing the results with PBPK computational models (Supplementary Methods). A comparison of the albumin concentrations for the experimental measurements and PBPK models of the data in the mixing chamber and each MPS compartment at day 2 (Q_sys_ = 5 ml/day, exchange rate 0.6 × total system volume per day), day 4 (Q_sys_ = 15 ml/day, 1.9 × total system volume per day), and day 6 (Q_sys_ = 30 ml/day, 3.8 × total system volume per day) are presented in Fig. 6. Not surprisingly, with increasing systemic flow rate, albumin is distributed more uniformly among MPSs on the 4-way platform. Interestingly, this flow-escalation experiment also illuminates that increasing the systemic exchange rate to ~4 total volume exchanges per day does not appear to adversely affect liver function significantly, as the inferred albumin secretion rates do not change with increased systemic medium exchange rates.

**Figure 7:**
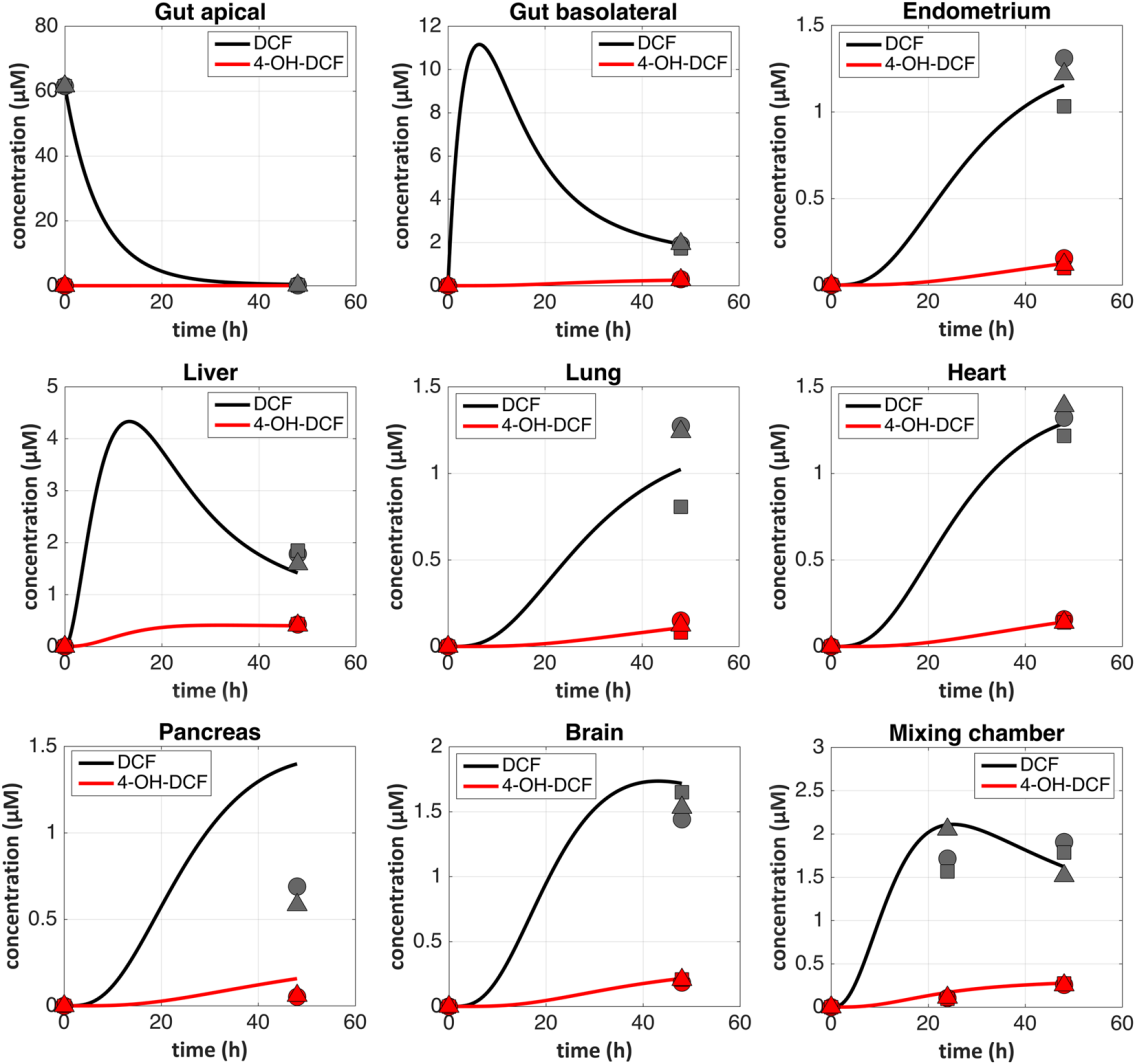
Quantification of diclofenac pharmacokinetics in 7-MPS platform. Samples for diclofenac (DCF) PK were collected from each compartment and quantified for parent drug (DCF, black symbols) and its primary metabolite (4-OH-DCF, red symbols). Individual points represent measurements from different platforms. The results were analyzed with PBPK models, illustrated by solid curves spanning the 48-hour experimental duration.

As a case study to demonstrate applicability of the platform and QSP framework to pharmacology, we investigated diclofenac (DCF) *in vitro* pharmacokinetics in the 7-MPS platform. First, PBPK model simulations (data not shown) of DCF distribution across MPSs –incorporating planned operational parameters (MPS volumes, flowrates) and previously measured values of DCF clearance (liver) and permeability (gut) in isolated MPSs – were used to predict that a DCF dose of 60 uM applied to the apical gut MPS (mimicking oral administration) would achieve a C_max_ in the platform mixer similar to that measured clinically (2–6 µM^55^). The experimentally observed media concentrations of DCF and its metabolite 4-OH-DCF in each MPS compartment (at 48 hours) and the mixing chamber (at 24 and 48 hours) following administration of this dose are illustrated in Fig. 7 (symbols); continuous model simulations for the entire 48 hours are shown with curves. The simulated curves resulted from the same model used to select dose, but instead of using clearance parameters from isolated liver MPS experiments we estimated values of DCF unbound intrinsic clearance and fraction of metabolism giving 4-OH-DCF specific for this experiment by fitting all 57 measured data points simultaneously. All other model parameters were fixed at experiment values (Table S3). As seen by following the magnitude and dynamics of distribution across the platform, the characteristics of the measured concentrations in each compartment are consistent with predictions of the PBPK model: the 60 µM initial concentration of DCF on the apical side of the gut resulted in a transient increase in the basal compartment to ~11 µM at 15 hr, which propagates to a transient increase in the liver to about 4 µM, followed by decline to the measured value of 2 µM by 48 hr as the concentration of the metabolite 4-OH-DCF increases. In turn, the mixer, just downstream of the liver, shows a peak of ~2 µM at 24 hr, consistent with our desired C_max_, then a slow decline over the next 24 hr as DCF is converted to 4-OH-DCF. The concentrations in the downstream MPSs rise later, with all MPS media approaching a similar concentration by the 48 hr endpoint as the media becomes more well-mixed during the experiment. The 4-OH-DCF metabolite, which is produced by cytochrome p450 enzymes present mainly in the liver MPS, circulated throughout the 7-MPS platform and was detected in all the other MPS compartments. The DCF unbound intrinsic clearance (CL_int(u)_) referring to hepatic metabolism was estimated to be 13.9 µL/min and approximately 19% of this clearance was estimated as formation of the 4-OH-DCF metabolite (the rest towards other, unmeasured, metabolites).

## Discussion

A range of different multi-MPS platform technologies are emerging to address the increasing need for analysis of complex human physiological responses in a controlled *in vitro* format, for applications ranging from disease modeling to testing efficacy and safety of therapeutic interventions^30–36,53,56,57^. The major contributions of the work presented here include a novel combination of platform performance attributes and integration techniques, functional longevity of up to 10 complex interacting MPSs, and demonstration of how QSP computational approaches can be combined with platform technologies to model distribution of both endogenously-produced molecules as well as *in vitro* pharmacokinetics. Together, the results provide a foundation for applying this platform to aspects of disease modeling where inter-MPS communication between a variety of different types of MPS constructs is needed, especially for long-term (weeks) culture experiments.

This platform combines several performance features in a novel manner, including: open system architecture; on-board, programmable and reconfigurable pumping that combines high flow rate capacity (0.05–300 uL/min), high DOF, and reliability over weeks of operation; relatively inert materials of construction; ability to accept off-the-shelf standard MPS constructs as well as custom designs; and standard multi-well plate footprint for use in standard incubators. Many current multi-MPS systems employ a closed-system fluidic format associated with traditional microfluidic chips, which operate in linear or circular flow schemes with very small fluid volumes, can have cost advantages when using expensive cells or reagents, and are amenable to *in situ* imaging and producing physiological shear rates on cell monolayers^35,58–60^. Closed-system microfluidic single- and multi-MPS platforms are commonly fabricated from PDMS^34,61–63^ as it has excellent oxygen transport, optical clarity, and prototyping properties, but also undesirable drug partitioning and hormone adsorption properties^64–66^. The drawbacks of PDMS have stimulated fabrication of MPS microfluidic platforms from materials more favorable for quantitative pharmacology applications^32,56,67,68^ with tradeoffs in reduced oxygen transport for supporting highly metabolically-active MPSs. Surface-modification approaches to reduce absorption of drugs into PDMS devices have also been described as successful in some implementations, though the strong tendency of PDMS for surface rearrangement can limit the length of time surface modification remains effective^65,66^.

Open microfluidics systems are a less common format, but design principles for these systems are emerging^69^ as they offer several advantages. Because gas exchange can occur at the air-liquid interface in an open system, the platform material itself does not need to be oxygen permeable. We built the fluid handling portion of the platform from polysulfone (PSF), a rigid, amber colored, machinable thermoplastic with food grade FDA approval (21CFR177.1655) and USP Class VI biocompatibility. Polysulfone is resistant to a wide range of chemical solvents, can be autoclaved, and is commonly used for instrumentation and medical devices. Polysulfone has dramatically lower surface adsorption and no bulk absorption of hydrophobic and lipophilic compounds, compared to elastomers like PDMS^70^.

Moreover, in the open system format, the various MPSs are highly modular and can easily be changed during operation, allowing replacement of individual MPSs as part of planned experimental protocols, e.g., to study effects of a “diseased” MPS (such as fatty liver) on “healthy” MPSs (or vice versa), or to do an “MPS transplant” to replace an MPS damaged by a perturbation (e.g. drug treatment) in an otherwise healthy platform. This feature was demonstrated in the 7-way experiments, where the pancreas MPS was replaced after 12 days based on prior data from static (off-platform) MPSs indicating decline of function over this time; subsequent analysis of c-peptide production indicated the on-platform performance of the pancreas MPS exceeded that of static culture by this metric. The open system format also allows access to all MPS compartments (e.g. apical and basal gut compartments) for manual or robotic fluid sampling and *in situ* measurements (e.g. TEER, imaging beat frequency) throughout operation. In addition, linking between MPSs does not have to be linear or circular, which, as noted, is an inherent limitation of many closed systems. The open system platform designs described here are also highly robust toward maintenance of sterility during operation and handling, as evidenced by continuous operation for over 4 weeks (i.e., the 4 weeks of co-culture and additional pre-culture of the liver compartment), including handling for collection of >1200 individual fluid samples.

An especially important feature of this multi-MPS platform is the incorporation of a high DOF, high capacity, long-lifetime on-board pumping system. On-board pumping saves dramatically on space and cost compared to commercial syringe or peristaltic pumps (e.g., the 7-MPS system has 36 DOF which operate the functional equivalent of 17 syringe pumps per platform), is more scalable, and allows systemic recirculation operation with low dead volumes (<1% of total volume). Each pump has a capacity of 0.05–300 µL/min and operates at initial calibration values over millions of cycles, with reliable function for at least 4.5 weeks as shown in the 10-way experiment.

This high DOF enables internal MPS recirculation flow rates, which govern oxygenation and local molecular transport *within* each MPS, to be uncoupled from flow rates associated with systemic exchange *between* MPSs in the recirculation loop connecting the MPSs together. Thus, in the liver compartment, internal mixing continuously perfuses medium through a scaffold containing an array of microscale 3D liver tissue, replicating the conditions used in Liverchip^®^ to measure drug PK, maintain an appropriate oxygen tension drop across the tissue, and support disease models^20,43,46,52,71^. Similarly, internal MPS recirculation facilitates mixing and molecular transport at the base of Transwell^®^ MPSs such as the gut/immune MPS. This deterministic mode of pumping, combined with mechanism-based QSP models, can enable accurate analysis and prediction of of endogenous and exogenous compound distribution. High DOF control also makes it easy to reconfigure this platform for new MPSs in either Transwell^®^ or microperfused (i.e., Liverchip^®^-like) formats as demonstrated by incorporation of the flow-through pancreas model on the 7-way. We have also shown the ability to dynamically exclude wells and change total system volume by using our 4-MPS platform for 2-MPS gut-liver interaction studies^30,36^. This high DOF pumping scheme with internal MPS recirculation distinguishes the platform described here from other multi-MPS platforms, which do not appear to have internal MPS recirculation pumps in addition to systemic exchange^31,33–35^.

The ultimate performance of any multi-MPS platform relies on the functional behavior of individual MPSs contributing to the integrated circuit. Arguably, one of the greatest challenges in the MPS field is “validation” of individual MPS functionalities and establishing reproducibility of MPS functions by different groups of investigators. Cell sources (e.g. primary postnatal, iPS-derived, or immortalized cell lines, homotypic vs heterotypic culture), physical microenvironment (ECM, 2D vs 3D, scaffold properties, mechanical forces), culture media, and device design (molecular transport, oxygenation, etc) all influence the performance of an individual MPS for a given application – and for any given MPS, multiple applications often exist, each with their own performance metrics. We thus designed this multi-MPS platform to accept two types of commercially-available MPS formats: Transwells^®^ and LiverChip^®^. As for several of the MPS categories included in the work, well-established MPS protocols for these standard formats could be imported to the platform for these proof-of-principle studies involving several existing MPS categories, and these formats were amenable to creation of functional MPSs in other categories. We also employed QSP to design experiments and interpret data, as QSP can account for variations in operational differences of individual MPSs, such as changes in volume and flow partitioning.

The liver MPS offers an illustrative case for functional scaling via QSP. The large diversity of intrinsic metabolic capacity *in vitro* for different published liver MPSs underscores the challenges in scaling multi-MPS interactions based on relative *in vivo* tissue masses or cell numbers, and motivates our analysis by QSP approaches that take into account these intrinsic variations. Here, we used commercially-sourced primary human cryopreserved hepatocytes and Kupffer cells cultured in a 3D, microperfused MPS modeled after the Liverchip^®^, as our lab and other laboratories have demonstrated that this format fosters retention of hepatic metabolic and secretory functions over weeks in culture. Moreover, this microperfused liver MPS has been well-characterized for drug PK and hormone kinetics using multiple drugs and donors^20,43,46,52,71^, and it has also been extended to gut-liver interactions^30,36^. However, within a given culture configuration, primary human hepatocytes from different donors exhibit quite significant variations in drug metabolism rates^20,43,72,73^. Further, connection to a gut/immune MPS^30,36^ or inflammation^20^ alters CYP450 activity of primary human hepatocyte-Kupffer cell co-cultures. Other higher-order multi-MPS platforms used HepG2/C3A in monolayer culture, iPS- derived hepatocytes, the HeparG hepatocyte cell line co-cultured stellate cells in spheroids, and primary human hepatocytes co-cultured with stellate cells and Kupffer cells^31,33–35^, but experimental reports of quantitative drug metabolism rates and *in vitro*-*in vivo* translation are as yet not available for these systems. Hepatocellular CYP450 expression and activity is typically much lower in 2D culture than 3D culture^44,50,74^; however, diffusion limitations can lower apparent metabolic rates in both 2D^75^ and 3D culture^44^. These variations in donor cell metabolic activity, configurational, and operational factors all together motivate the QSP approach, which takes such variations into account.

A similar analysis of how to assess performance of all the other MPSs from the perspective of scaling is beyond the scope of this discussion, but observations about performance of a few particular MPSs highlight some of the additional issues and noteworthy findings from this work. The brain MPS used in the 7-way was adopted from protocols developed by the Thomson and Murphy labs at University of Wisconsin, Madison^22^, with laboratory exchanges confirming that comparable results for performance metrics were obtained in the two different geographic locations. This outcome is arguably an important confirmation of reproducibility across independent laboratories and geographic locations. Interestingly, the brain MPS exhibited improved performance metrics during interaction on the platform compared to isolation as assessed by production of the metabolite NAA. The mechanisms underlying this observation are as yet unknown, but may involve enhanced molecular transport or factors produced or consumed by other MPSs.

Underscoring the significant infrastructure needed to implement higher-order multi-MPS platforms, we substituted a commercial iPS-derived brain model for the 10-MPS experiment because its adult-derived nature and shorter maturation time facilitated workflow coordination to mature MPSs off platform in tandem. Also, the brain MPS configurations employed here lacked an explicit blood-brain-barrier (BBB), as BBB transport was not a focus of the drug studies analyzed here. However, the versatility of the platform design does not preclude accommodating an explicit BBB model from among many that have been described^76^, for applications where this feature is necessary. A similar analysis of the balance between performance, complexity, and cost as considerations in the context of particular applications can be made for other MPSs.

The endometrium MPS illustrates additional trade-offs between complexity and function. The most common format for epithelial barrier MPSs, such as the lung and gut MPSs employed in this work, involve epithelial monolayers plated on Transwell^®^ inserts using well-defined protocols^48,77–79^. A more recent microfluidic epithelial culture barrier model developed by Huh and coworkers employs a porous PDMS membrane to support the epithelial layer, and is frequently combined with endothelial cells on the alternate side of the membrane^63,80^. The human endometrium is characterized by complex epithelial-stromal interactions, hence we developed and implemented a more complex model with epithelial cells on top of a 3D stromal layer, created by encapsulating the stromal cell line in a synthetic extracellular matrix (ECM) tailored to support the tissue integrity and function in long-term (4 week) culture^81,82^. The relative scarcity and variability of primary endometrial cells made the choice of cell lines more practical for these proof-of-principle studies, but we have demonstrated that this 3D epithelial barrier culture approach can be successfully applied to primary human endometrial cells^81,82^. Recent advances in expansion of primary human endometrial cells^83,84^ will make studies with primary endometrium – as well as other similarly finicky epithelial cells^85^ – more feasible in the future.

Another noteworthy finding from these multi-MPS interaction studies is that basic MPS functions can apparently be maintained for weeks in interaction mode, with constant systemic circulation of the media among MPSs. In our model, we loaded each MPS on the platform in its specialized maintenance medium, operated the platform under systemic circulation conditions for two days, and then exchanged the medium within each MPS with fresh MPS-specific medium and the mixer with the common medium. (The fluidic channels, representing <1% of the total volume, were not exchanged). The standard systemic circulation rate we used of 0.5–1 total medium volumes per day is comparable to other multi-MPS platforms described in the literature^30–36,53,56,57^. Most MPSs showed at least comparable phenotypic metrics on the platform compared to off-platform (Figs 3, 4, 5 and S4–6). Notably, during platform interaction studies, the liver MPS converted a parent drug, DCF, to a known primary metabolite (4-OH-DCF) in a pharmacologically-relevant manner, indicating maintenance of CYP450 activity and suggesting that operational factors such as culture medium oxygenation are suitable. For the pancreas MPS and the brain MPS, which both showed improved metrics for a single representative phenotype viability (c-peptide and NAA production, respectively) on the platform compared to in isolation off-platform, a possible explanation is the presence of convection to enhance nutrient exchange on platform, compared to static conditions in isolation. However, we cannot rule out that factors produced or consumed by other MPSs on the platform influenced the observed function of the pancreas and brain MPSs. Encouragingly, a 6-fold increase in the systemic flow rate (i.e., a 6-fold increase in the exchange rate among MPSs compared to the standard rate) did not appear to have deleterious effects on MPS function; for example, albumin production was unaffected by this 6-fold escalation of exchange rate for several days (Fig. 6). These results add to the accruing data on how multi-MPS interactions can have both positive and negative effects on individual MPS function.

Finally, this work demonstrates how a QSP approach can be applied to plan and interpret pharmacological experiments in multi-MPS platforms, which becomes increasingly challenging as the number of MPSs increases. QSP approaches using PBPK models of our platforms were critical to identifying operational strategies for experiments (drug dosing, sampling time points, inter- and intra-MPS media flowrates, etc.) to obtain informative results. By comparing our experimental results with the theoretical distribution values calculated from the PBPK models in the studies of endogenous albumin distribution in the 4-MPS platform and exogenous oral drug distribution in the 7-MPS platform, we demonstrated that our multi-MPS platforms operate deterministically. This predictable and calculable platform operation enables the study of PK/PD (cue-response) relationships on such platforms in the future. Further, having models that capture the dynamics of molecular distribution throughout the platform means we can simplify experimental operation by collecting samples from the mixer only, and predict drug concentrations in the other compartments.

The experiments described here are proof-of-principle in nature, intended to demonstrate the capabilities of the platform technology and QSP approaches. While the technology is robust up to 10-way interactions, such substantial resources are required to operate in a 10-way mode (experimental design, MPS preparation, analysis of samples, etc.) that it is unlikely the technology will be widely deployed for such higher-order interactions in the near future. However, we anticipate that the platform technology is highly amenable to illuminating specific biological or pharmacological questions involving lower-order (2–4) interactions. We have recently deployed a 2-way version of this platform to show cross-regulation of the liver MPS metabolic function in gut-liver interactions during baseline and inflamed conditions^30,36^. These studies provide a foundation for ongoing work examining 4-way interactions between gut, pancreas, liver, and brain in various chronic diseases such as Parkinson’s^86^, where QSP approaches aid conceptualization of the platform configuration and scaling of MPSs. An outstanding question in both single and multi-MPS formats is how to replace the essential functions of missing MPSs (*e*.*g*. endocrine, *etc*.) for any given physiology or disease model, and technologies are emerging to address this need^87^.

In summary, we have demonstrated a generalizable approach to linking MPSs within a fluidic platform to create a physiome-on-a-chip approach capable of generating complex molecular distribution profiles for advanced drug discovery applications. This adaptable, reusable system has unique and complementary advantages to existing microfluidic and PDMS-based approaches, especially for applications involving high logD substances (drugs and hormones), those requiring precise and flexible control over inter-MPS flow partitioning and drug distribution, and those requiring long-term (weeks) culture with reliable fluidic and sampling operation. We anticipate this platform can be applied to a wide range of problems in disease modeling and pre-clinical drug development, especially for tractable lower-order (2–4) interactions.

## Methods

### Platform Fabrication and Assembly

Device layers were designed in CAD and commercially machined. Pneumatic plates were machined in acrylic and solvent bonded to form two-layer manifolds, while fluidic plates were machined from monolithic polysulfone stock material. Pneumatic plates were post-processed with vapor polishing and fluidic plates were cryo-deburred to remove sharp burrs. The PU membrane was supplied by American Polyfilm Inc. and mounted onto grip rings (Ultron Systems UGR-12) to provide uniform tension. Mounted membranes were laser cut to remove material around screw holes, then sterilized using ethylene oxide gas.

Sterilized layers were assembled in a laminar flow hood, and the cell culture area of the device was covered with a standard multi-well plate lid. Screws, washers, and pneumatic tubing could then be inserted under non-sterile conditions. Prior to culture, platforms were primed overnight by filling the wells with 1% bovine serum albumin in PBS and running all pumps to passivate fluid-contacting surfaces. After priming, the fluid was aspirated and cell culture media was added to each well at the appropriate volumes (see Table S1). Sampling and media changes were performed every 48 hours by manually collecting and replacing the media. A GUI software interface was used to set flow rates and calibration adjustments, while also monitoring pressure and vacuum during the experiment.

### MPS Preparation

#### Liver MPS

The liver MPS was prepared as previously described^20,43^. LiverChips^®^ were assembled 4 days prior to experiment start and primed overnight with 1% BSA and Pen/Strep in PBS. Cryopreserved human primary hepatocytes and Kupffer cells (Life Technologies, HMCPMS and HUKCCS) were recovered according to supplier’s instructions. Polystyrene scaffolds (CN Bio Innovations) were coated with rat tail collagen I (BD 354236). Cell suspensions (10:1 HMCPMS: HUKCCS) were seeded (6.6 × 10^5^ cells per scaffold) into scaffolds housed in the platform or LiverChip^®^ in cold hepatocyte seeding medium (Advanced DMEM + Cocktail A + FBS). After 24 hours, the media was changed to pre-culture media (Advanced DMEM + Cocktail B). On the third day, the medium was changed to Williams E medium + Cocktail B + 100 nM hydrocortisone and the experiment commenced.

#### Gut MPS

Gut MPS was prepared as previously described^30^. Briefly, Caco-2 (4-MPS system) or C2BBe1 (7- and 10-MPS system) along with HT29-MTX-E21 cells (Sigma) were seeded onto rat tail collagen I- (Corning 354236) coated Transwell^®^ inserts (Corning 3460) in a 9:1 ratio (1 × 10^5^ cells/cm²) in 500 µL seeding medium. The top and bottom compartments of the Transwell^®^ plate were fed with 500 µL and 1.5 mL of seeding medium respectively every 2–3 days. After 7 days, cells were switched to serum-free culture medium. The immune component of the gut MPS was comprised of dendritic cells differentiated from frozen stocks of Leukopak aliquots (STEMCELL Technologies 70500). Cells were thawed then isolated using the EasySep Human Monocyte Enrichment Kit (STEMCELL Technologies 19058) and differentiated in dendritic differentiation medium. After 7 days (day 20 of gut insert maturation), immune cells were harvested using Accutase (Gibco™ A11105-01) and seeded on to the basal side of the inverted gut Transwell^®^ in gut basal medium. After 2 hours, cells were returned to culture plate and fed with gut basal medium and gut apical medium in the basal and apical compartments, respectively.

#### Lung MPS

Cryopreserved primary normal human bronchial epithelial (NHBE) cells (Lonza) were seeded at a density of 1.5 × 10^5^ cells/cm^2^ on human placental collagen IV (Sigma-Aldrich) coated Transwell^®^ inserts (Corning 3470). Cells were cultured in lung medium^77^ until confluence (2–3 days), and then maintained at the air-liquid interface with basal media changes every 2 days for three weeks to induce differentiation into ciliated and secretory cell populations.

#### Endometrium MPS

The endometrium MPS, a co-culture of Ishikawa human endometrial epithelial adenocarcinoma cells (Sigma-Aldrich) and hTERT-immortalized human endometrial stromal cells (tHESCs) (ATCC), was prepared as previously described^81^. Briefly, polyethylene glycol (PEG) hydrogel matrices comprising 5 wt/wt% PEG vinyl sulfone (PEGVS) (JenKem Technology, Beijing) functionalized with 2 mM total adhesion/matrix-binding peptides (Boston Open Labs, Cambridge, MA), 4.2 × 10^6^ tHESCs/mL, and 1.9 mM crosslinking peptide MMP-CL in 1x PBS, 1 M HEPES buffer (pH 7.8)) were fabricated in Transwell^®^ inserts (Corning 3470). 24 h after initiation of stromal cultures, Ishikawa cells were harvested via trypsinization, resuspended in DMEM/F12/FBS and seeded at a density of 75,000 cells/Transwell^®^ (225k cells/cm^2^) on top of gel-encapsulated stromal cells. Apical medium was changed 24 h after seeding to remove non-adherent epithelial cells, and constructs were cultured for an additional two days prior to the start of the experiment.

#### Brain MPS for 7-way

Polyethylene glycol (PEG) hydrogels were prepared according to a previously published protocol^22^. Briefly, PEG hydrogels were prepared fresh, using 40 mg/mL 8-arm PEG-norbornene (Jenkem A10037-10) in 2x PBS containing KCGGPQGIWGQGCK peptide (ThermoFisher) at 60% cross-linking density (~19.2 mM), 2 mM CRGDS C-amidated peptide (ThermoFisher), and 0.05 wt% Irgacure I2959 (Sigma-Aldrich 410896) initiator. Gels were polymerized in Transwell^®^ inserts (Corning 3470) with 40 µL total volume and 4.8 J/cm^2^ of 365 nm UV light (10 minutes, 8 mW/cm^2^).

Neural progenitor cells (NPCs) derived from the human H1 ES line were obtained as a gift from James Thomson, Morgridge Institute, Madison, WI. The NPCs were cultured in DMEM/F12 (Gibco 11330) supplemented with 220 µM L-ascorbic acid (Sigma-Aldrich A8960), 80 nM sodium selenite (Sigma-Aldrich S5261), 6.5 mM sodium bicarbonate (Sigma-Aldrich S5761), 5 ng/mL human FGF-2 (Fisher Scientific 507515502), and N-2 and B27 supplements at 1X final concentration (Life Technologies 17502-048 and 17504-044). NPCs were maintained below 90% confluence on matrigel-coated polystyrene (Corning 354230) and harvested for MPS seeding with Accutase (ITC AT104) between passages 4–6. PEG hydrogels were swelled in DMEM/F12 for 12–24 hours and then seeded with NPCs at a density of 50,000 cells/Transwell^®^ in fresh NPC media. Neural constructs were cultured for 12–14 days to allow for expansion and differentiation of neuronal and glial subpopulations, as confirmed by increased immunostaining for neural marker β3-tubulin and astrocyte marker GFAP. NPC media was changed every 48 hours, using 200 µL in the apical compartment, and 1 mL in the basal compartment.

#### Brain MPS for 10-way

CNS cells were obtained from Axiogenesis (CNS.4U, Cat #Ax-C-HZ02-2M) as a cryopreserved mixture of iPS-derived astrocytes and neurons (glutamatergic, GABAergic, and dopaminergic). Three days prior to interaction, Transwells^®^ (24-well, 0.4 µm pore size) were matrigel coated (0.1 mg/ml in PBS) for one hour at 37 °C, then seeded with 200,000 freshly thawed cells. Cells were seeded and cultured in Neuro.4U media (Cat #Ax-M-NBM250) with DA supplement (Cat #Ax-M-DCS-DA) according to the manufacturer’s recommendations. Constructs were transferred to platforms 2–4 days post-seeding. On-platform, apical media remained Neuro.4U + DA, while basal media was replaced with Common Media.

#### Heart MPS

Human iCell Cardiomyocytes 2 (Cellular Dynamics International, Inc CMC-100-012-000.5) were seeded at a density of 0.3 × 10^6^ cells/cm^2^ onto human fibronectin coated Transwell^®^ inserts (Corning 3470). Cardiomyocytes were cultured in plating media (CDI CMC-100-010) for 48hrs to recover and changed to maintenance media (CDI CMM-100-120) for an additional 48hrs prior to the start of the experiment.

#### Pancreas MPS for 7-way

Rat pancreatic islets were obtained from the Joslin Diabetes Center (Boston, MA), isolated according to standard isolation protocol^88^. Islets were delivered on ice, filtered upon arrival to achieve a size range of 70–140 µm diameter, and maintained in a non-tissue culture treated petri-dish with 20 mL of RPMI media (Thermo Fisher, 72400047) supplemented with 0.125% BSA (Sigma, A9576) and 1% Penicillin-Streptomycin.

Three days before platform experiments began, 75 islets were handpicked under a stereo microscope and seeded into a polystyrene scaffold (CN Bio Innovations). The scaffold was sandwiched between two porous membranes and held in place with retaining rings (CN Bio Innovations). The entire membrane-scaffold sandwich was contained within the custom-made removable insert (see platform section).

#### Pancreas MPS for 10-way

Rat pancreatic islets were obtained from the Joslin Diabetes Center (Boston, MA), isolated according to standard isolation protocol^88^. Islets were delivered on ice, filtered upon arrival to achieve a size range of 70–140 µm diameter, and maintained in a non-tissue culture treated T75 flask with 10 mL of RPMI media (Thermo Fisher, 72400047) supplemented with 0.125% BSA (Sigma, A9576) and 1% Penicillin-Streptomycin.

Two days before platform experiments began, islets were spun down at 1000 rpm for 3 minutes, washed twice with Ca-free PBS (centrifuging after each step) and then resuspended in 1.5% alginate (Novamatrix, SLG20) in deionized water in a non-tissue culture treated petri-dish. Islets were then handpicked under a stereo-microscope for seeding. 25 islets (in 20 µL total volume) were seeded into each polyester Transwell^®^ insert with 8 µm pore size and 33 mm^2^ surface area (Corning, 3464). After seeding, 20 µL of 20 mM BaCl_2_ solution (20 mM BaCl_2_-2H_2_O, 2 mM KCl, 10 mM HEPES, 268 mM Mannitol, all from Sigma) was added to each Transwell^®^. The alginate was allowed to crosslink for 5 minutes at 37 °C then the Transwells^®^ were washed three times with pre-warmed RPMI. Finally, 800 µL of RPMI was added to each well with 200 µL added inside the insert for a total of 1000 µL media volume per well.

#### Muscle MPS

Primary Human Skeletal Muscle Myoblasts (Lonza CC-2580) were seeded at a density of 0.24 × 10^6^ cells/cm^2^ onto Human Fibronectin (Gibco 33016015) coated Transwells^®^ (Corning 3470) in SkGM™-2 Skeletal Muscle Cell Growth Medium-2 (Lonza CC-3245). Transwell^®^ inserts contained 150μL apical volume and 1 mL basal volume After 24 hours, myoblasts were changed to differentiation media (DMEM-F12 with 2% Horse serum) for an 4 days, changing media every 48 hours, and subsequently returned to SkGM™-2 Skeletal Muscle Cell Growth Medium-2 at the start of the experiment.

#### Skin MPS

The *in vitro* skin model was purchased from EpiSkin (Lyon, France) and consists of reconstructed human epidermis from normal human keratinocytes cultured on a collagen matrix at the air-liquid interface. The EpiSkin kit (EpiSkin Large model 1.07 cm^2^) was shipped at Day 13 of maturity in nutrient agar. Upon receipt of the EpiSkin kit, the inserts were transferred from their nutrient agar under aseptic conditions into a sterile culture multi-well plate containing 2 ml per well of pre-warmed differentiation medium (L’Oreal DIFF + ). Medium was replaced every 48 hours.

#### Kidney MPS

Human Renal Proximal Tubule epithelial cells (RPTEC) were plated in RPTEC growth media- DMEM:F12 supplemented with EGF (10ng/mL), Hydrocortisone (36ng/mL), Triiodothyrine (2 pg/mL), ITS (1:1000), Pen/Strep (100 U/mL) onto human placental Collagen IV (Sigma Aldrich cat.# C5533) coated Transwells^®^ (Corning 34130) at a density of 0.075 × 10^6^ cells/ cm^2^. Transwell^®^ inserts contained 200μL apical volume and 1 mL basal volume. Full apical media volume and half basal media volume were replaced every two days.

### Platform Operation and Sample Collection

Mature constructs were transferred to the platform and cultured for the duration of the experiment in multi-MPS interaction. Basal and apical media was changed every two days, with samples collected from each compartment and stored at −80 °C.

Table S1 outlines the compartmental volumes and flow rates used in all the experiments. The systemic flow rate (output from the mixer) was computationally determined to enable proper mixing of endogenously produced biomolecule and exogenously added drugs. The computational methodology was adapted from our previous work^38^ and is described in Supplementary Materials. The flow partitioning from the mixer to each MPSs was scaled based on physiological cardiac output to each tissue type in human.

### MPS Metric Measurements

#### Liver MPS

Samples from each compartment were assayed for albumin using a human-specific ELISA (Bethyl Labs E80-129).

#### Gut MPS

Barrier integrity was quantified by TEER using the EVOM2 and the Endohm-12 (World Precision Instruments) at 37 °C.

#### Lung MPS

Lung cultures were incubated for 2 hours with 150 µL 1 × PBS in the apical compartment. After removal of wash buffer, barrier integrity was quantified by TEER using the EVOM2 and the Endohm-6 (World Precision Instruments) at 37 °C.

#### Endometrium MPS

Secreted IGFBP-1 protein was quantified by ELISA for IGFBP-1 (R&D systems DY871)^81^. Manufacturer methods were adapted for 384-well plate (ThermoFisher 464718) to minimize required sample volume.

#### Brain MPS

N-acetylaspartate in samples from the apical side of the brain MPS was quantified by LC-MS/MS by Alliance Pharma. The analytical methods are described in detail in the supplementary material.

#### Heart MPS

Cardiomyocyte beating was monitored every four days using with a Leica DMI 6000 Microscope (37 °C, 5% CO_2_) and Oasis Surveyor software and recorded as videos using Open Broadcast software. Beat frequency was extracted from the video files using Matlab.

#### Pancreas MPS

C-peptide was quantified by ELISA (80-CPTRT-E01, ALPCO).

#### Muscle MPS

Myostatin was quantified by ELISA (DGDF80, R&D).

#### Skin MPS

TEER measurements were performed according to a protocol adapted from a publication by the L’Oreal Research group^89^. TEER was measured using the EVOM2 and the Endohm-12 (World Precision Instruments) and D-PBS (Ca2+, Mg2+) as an electrolyte solution. The day of TEER measurement, 0.3 ml of D-PBS was added onto the apical side of the insert and left to equilibrate for 15 min at 37 °C, and 2.5 ml of D-PBS were added inside an EndOhm Chamber. Following TEER measurements, D-PBS was removed from the apical side to restore the air-liquid interface.

#### Kidney MPS

TEER was measured using the EVOM2 and Endohm-6 chamber (World Precision Instruments) at 37 °C.

### Computational analysis of albumin and diclofenac kinetics

Computational investigations of the experimental output and graphical analyses were performed in Matlab R2016a (The MathWorks Inc., Natick, MA). The details of computational models are presented in the supplementary materials.

In brief, the experimentally observed albumin and DCF distribution kinetics data on-platform were analyzed with the aid of mechanistic *in vitro* PBPK models, the structure of which was guided by the MPS interconnection scheme and the physiological processes taking place on-platform. The developed mechanistic models utilized fixed values for operational and certain biological parameters, and were fitted to the experimental data (across all platform compartments and time points simultaneously) to estimate specific model parameters that represent processes of interest in each experiment: rate of production of albumin by liver MPS in the 4-way albumin distribution experiment, and pharmacokinetic processes (e.g. intrinsic hepatic clearance of DCF and the fraction converted to 4-OH-DCF by the liver MPS) in the 7-way DCF kinetics experiment. The details of computational models and their application are presented in the supplementary materials.

The analytical methods using mass spectrometry are described in detail in the supplementary material. For drug quantification, we developed a 3-in-1 assay, which can quantify diclofenac, 4-OH diclofenac, and hydrocortisone (results not shown) simultaneously.

## Electronic supplementary material

Supplementary Information

Video 1

Video 2
