# Supplementary material for: Interconnected Microphysiological Systems for Quantitative Biology and Pharmacology Studies
Source: Sci Rep. 2018 Mar 14;8:4530. doi: 10.1038/s41598-018-22749-0 (PMC5852083; doi:10.1038/s41598-018-22749-0)
Supplement: Supplementary file 1 — Supplementary Information [file 41598_2018_22749_MOESM1_ESM.doc]

# **Interconnected Microphysiological Systems for Quantitative Biology and Pharmacology Studies**

# Collin D. Edington^1,#^, Wen Li Kelly Chen^1,#^, Emily Geishecker^1^, Timothy Kassis^1,4^, Luis R. Soenksen^2,4^, Brij M. Bhushan^2,4^, Duncan Freake^5^, Jared Kirschner^5^, Christian Maass^1^, Nikolaos Tsamandouras^1^, Jorge Valdez^1^, Christi D. Cook^1,3^, Tom Parent^5^, Stephen Snyder^5^, Jiajie Yu^1^, Emily Suter^1^, Michael Shockley^1^, Jason Velazquez^1^, Jeremy J. Velazquez^1^, Linda Stockdale^1^, Julia P. Papps^1,3^, Iris Lee^1^, Nicholas Vann^1^, Mario Gamboa^1^, Matthew E. LaBarge^1^, Zhe Zhong^1^, Xin Wang^1^, Laurie A. Boyer^6^, Douglas A. Lauffenburger^1,3,6,8^, Rebecca L. Carrier^7^, Catherine Communal^1^, Steven R. Tannenbaum^1,8^, Cynthia L. Stokes^9^, David J. Hughes^10^, Guarav Rohatgi^5^, David L. Trumper^2,4*^, Murat Cirit^1,8*^, Linda G. Griffith^1,2, 3,8^*

^1^Department of Biological Engineering, Massachusetts Institute of Technology, Cambridge MA

^2^Department of Mechanical Engineering, Massachusetts Institute of Technology, Cambridge MA

^3^Center for Gynepathology Research, Massachusetts Institute of Technology, Cambridge, MA

^4^Research Laboratory of Electronics, Massachusetts Institute of Technology, Cambridge, MA

^5^Continuum LLC, Boston, MA

^6^Department of Biology, Massachusetts Institute of Technology, Cambridge MA

^7^Department of Chemical Engineering, Northeastern University, Boston, MA

^8^Center for Environmental Health Sciences, Massachusetts Institute of Technology

^9^Stokes Consulting, Redwood City, CA

^10^CnBio Innovations, Hertfordshire, United Kingdom

# equal contributions

*corresponding authors

Linda G. Griffith – [griff@mit.edu](mailto:griff@mit.edu)

David L. Trumper – [trumper@mit.edu](mailto:trumper@mit.edu)

Murat Cirit – [mcirit@mit.edu](mailto:mcirit@mit.edu)

**Supplemental Information**

**Description of pumping architecture**

Pumping is driven from outside the incubator by a microcontroller and a pneumatic solenoid manifold that controls 36 channels of tubing running through the back of the incubator to intermediary connectors. Inside the incubator, tubing is attached to the platform through valved breakaway couplings to allow easy removal from the incubator for media changes and sampling. The connectors and software architecture allow the setup to be compatible with both the 4-way and 7-way platforms, as well as many future platform variants, with minimal modification to the software configuration. Pump flow rates and calibration factors are set through a graphical user interface on a laptop, and can be sent to a customized microcontroller (National Instruments myRIO-1900) over USB or WiFi. Both manual and pre-programmed control of pump rates are available depending on the experimental needs, and the microcontroller can run independently of the laptop.

**CAD-based platform design**

The mechanical configuration of the platforms is created in a CAD program in a table-driven process. That is, building-block models for each MPS compartment are parametrically defined, and dropped into a plate drawing, and adjustments to fluid volumes and MPS heights are adjusted in a semi-automated manner by a table of target MPS parameters. Table-driven CAD dramatically reduces the time required to iterate on new formats and create designs ready for machining.

**Table S1**

MPS configurations, volumes, and flow rates of 4-, 7-, & 10-way platforms.

|  | **4-MPS Platform** | | | | **7-MPS Platform** | | | | **10-MPS Platform** | | | |
| --- | --- | --- | --- | --- | --- | --- | --- | --- | --- | --- | --- | --- |
| **Compartment** | **Basal Volume (mL)** | **Apical Volume (mL)** | **Recirc Flow Rate (mL/day)** | **Interaction Flow Rate (mL/day)** | **Basal Volume (mL)** | **Apical Volume (mL)** | **Recirc Flow Rate (mL/day)** | **Interaction Flow Rate (mL/day)** | **Basal Volume (mL)** | **Apical Volume (mL)** | **Recirc Flow Rate (mL/day)** | **Interaction Flow Rate (mL/day)** |
| **Mixer 1** | 3.50 | - | 86.40 | - | 3.50 | - | 86.40 | - | 2.50 | - | 1.00 | - |
| **Liver** | 1.60 | - | 86.40 | 1.50 | 1.60 | - | 86.40 | 1.30 | 1.60 | - | 1.00 | 1.60 |
| **Gut** | 1.50 | 0.50 | 21.60 | 2.51 | 1.50 | 0.50 | 21.60 | 3.80 | 1.50 | 0.50 | 0.25 | 3.60 |
| **Lung** | 1.00 | - | 21.60 | 0.50 | 1.00 | - | 21.60 | 0.50 | 1.00 | - | 0.25 | 0.46 |
| **Endometrium** | 1.00 | 0.10 | 21.60 | 0.50 | 1.00 | 0.10 | 21.60 | 0.70 | 1.00 | 0.10 | 0.25 | 0.46 |
| **Brain** | - | - | - | - | 1.25 | 0.20 | 43.20 | 2.60 | 1.25 | 0.20 | 0.25 | 2.80 |
| **Heart** | - | - | - | - | 1.00 | 0.15 | 43.20 | 0.90 | 1.00 | 0.15 | 0.25 | 1.10 |
| **Pancreas** | - | - | - | - | 1.60 | - | 43.20 | 0.20 | 1.25 | 0.20 | 0.25 | 0.30 |
| **Mixer 2** | - | - | - | - | - | - | - | - | 3.50 | - | 1.00 | - |
| **Kidney** | - | - | - | - | - | - | - | - | 1.00 | 0.10 | 0.25 | 4.30 |
| **Muscle** | - | - | - | - | - | - | - | - | 1.00 | 0.10 | 0.25 | 4.30 |
| **Skin** | - | - | - | - | - | - | - | - | 1.25 | - | 0.25 | 1.10 |
| **Sum Total** | 8.60 | 0.60 | 237.60 | 5.01 | 12.45 | 0.95 | 367.20 | 10.00 | 17.85 | 1.35 | 5.25 | 20.02 |

**Figure S1.**

Render of 4-MPS platform (left) and image of 10-MPS platform (right). The 10-MPS platform is comprised of a 7-MPS plate and a 3-MPS plate. Plates are fluidically linked using low dead-volume microbore tubing. A high flow rate between plates ensures that the mixing chambers of each behave as one well-mixed body of fluid. Pneumatic connections (left and center) deliver air and vacuum, and drive pumps across both platforms.


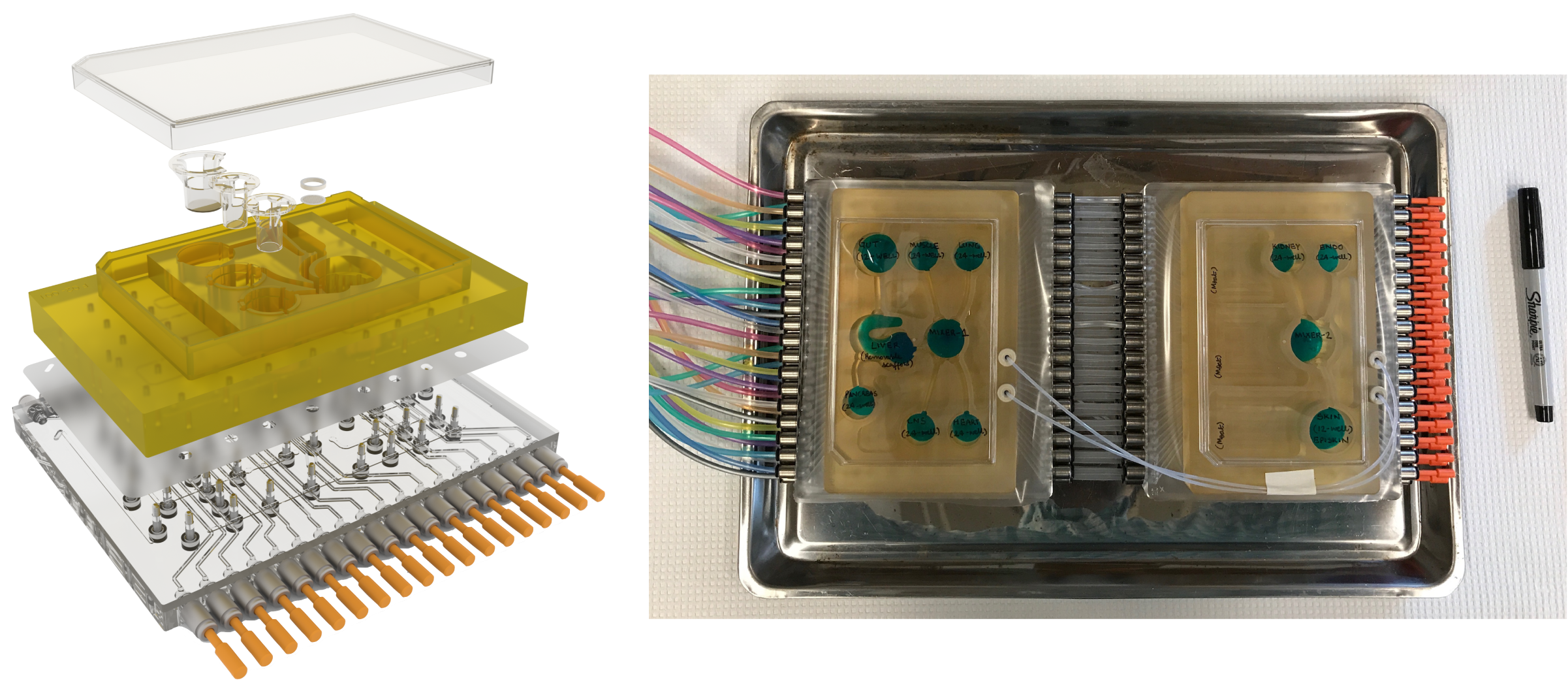


**Figure S2.**

Software rendering showing a cross-section of the 4-MPS platform to demonstrate theory of operation (a). Fluid is held in open wells similar to traditional cell culture methods, but transport is precisely controlled through integrated pumps beneath the wells. Each well represents a single MPS, and contains its own recirculation pump (shown in b). When fluid is added through the inlet, the level increases slightly and excess fluid leaves the well through the spillway (shown upper left in panel b). Shown in (c) is an expanded cross-sectional view of the peristaltic pumps, with the fluid path represented by a red line.


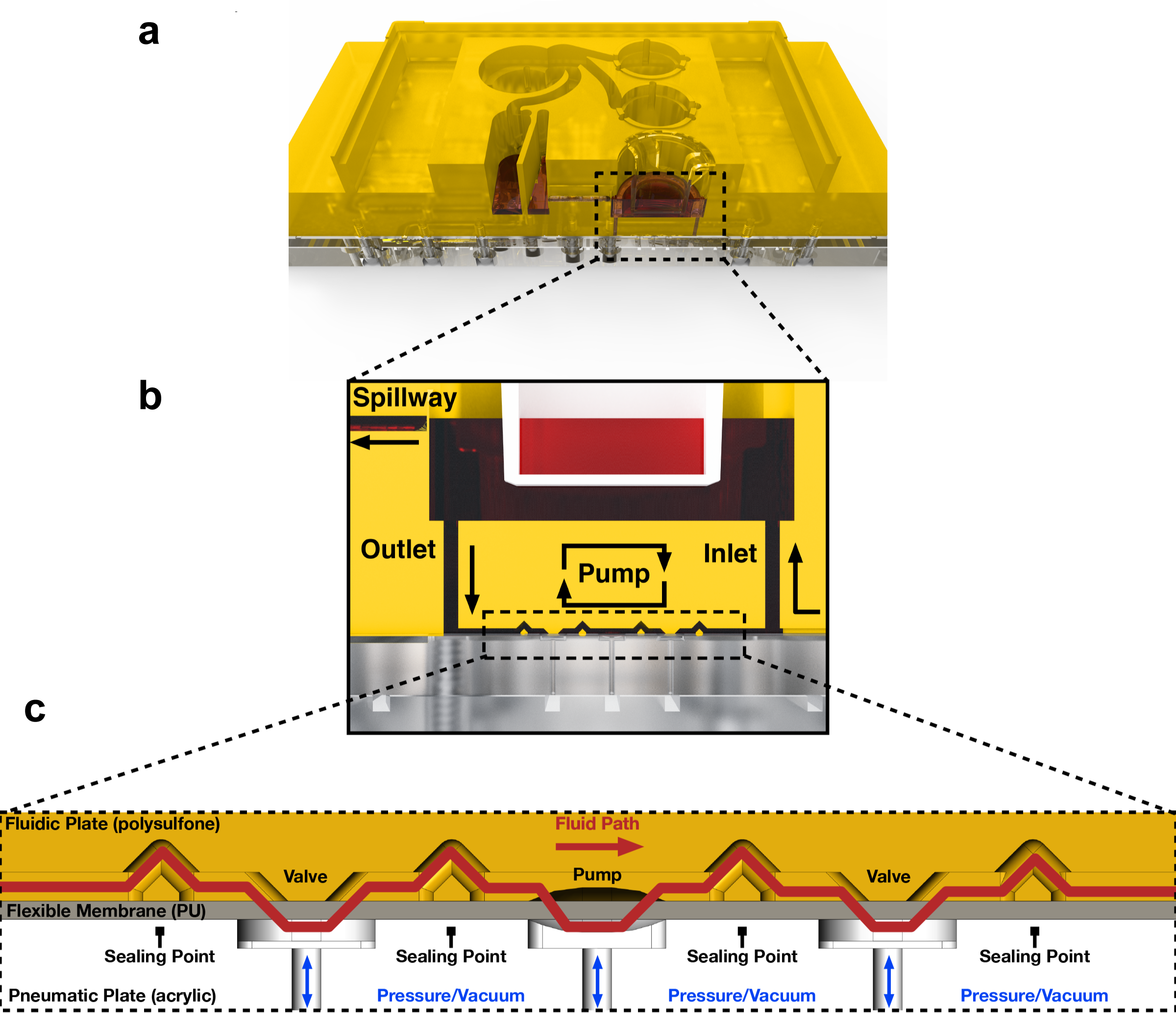


**Figure S3.**

(a) Flow rates of pumps in 4-MPS platforms when operating at 2 Hz. Each pump is designed for a 500 nL stroke volume, therefore 2 Hz operation should target 1 µL/s. Flow rate was determined from the time required to fill a capillary of known volume. Mean and standard deviation of all pumps on a single platform are shown for each platform in order to show levels of consistency both within and across platforms. (b) Flow rates of pumps in 7-MPS platforms when operating at 2 Hz, measured as described above. (c) Demonstration of pump rates from a single 7-MPS platform, before (black circles) and after (red squares) calibration using software adjustments. Calibration factors were calculated from the first set of measured flow rates. Each point is a single pump on the platform.

(d) Pump rates from the pairs of 7- and 3-MPS platforms used to create the 10-MPS system. Flow rates were measured as described above.


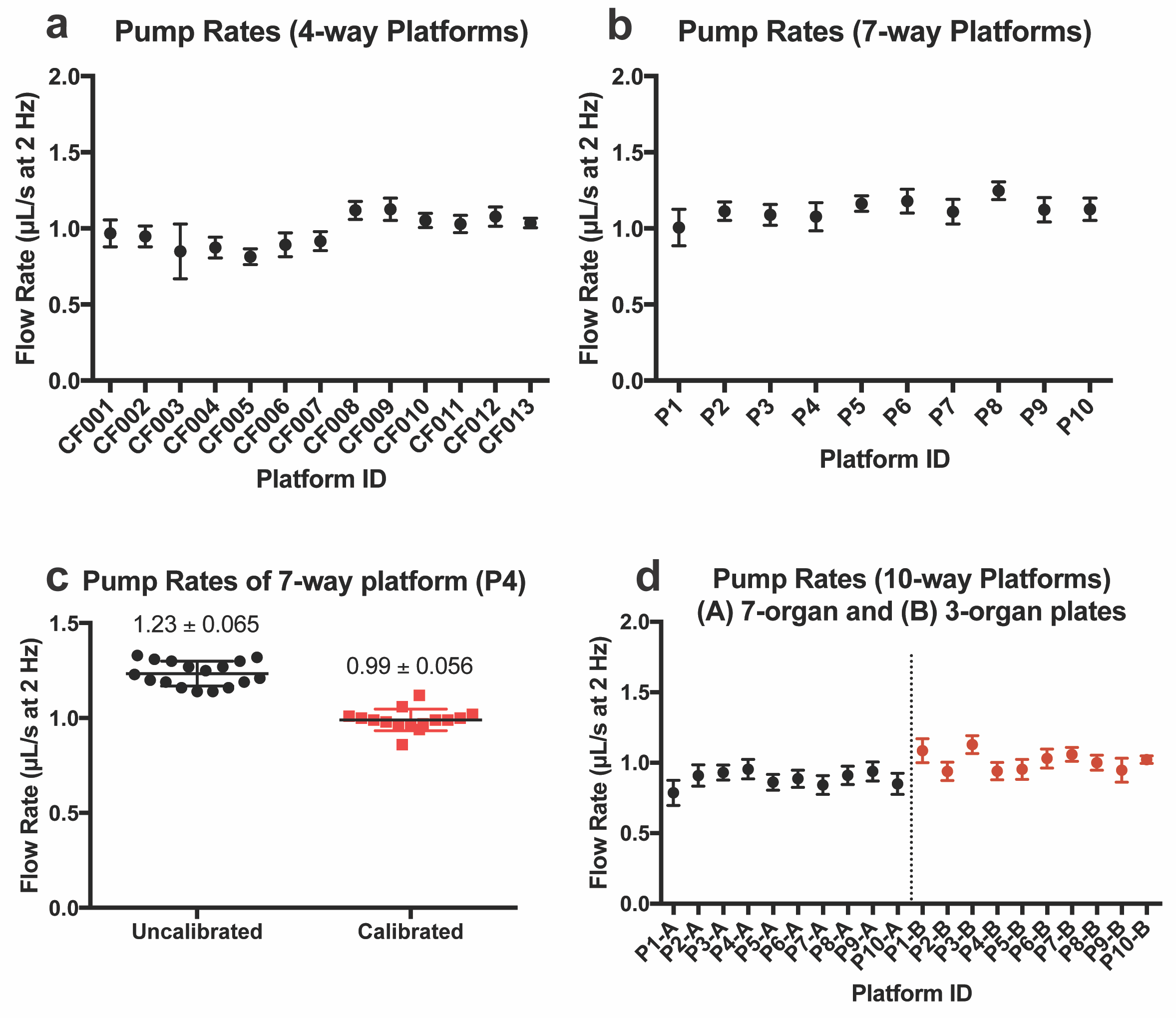


**Figure S4**.

Metrics of tissue function off-platform (i.e., in isolation) measured during a 2-week isolated culture of 4 different MPSs. MPSs representing gut, lung, and endometrium were cultured in isolated transwells as described in methods, and liver MPS was cultured in isolated flow using a LiverChip device as previously described (43). Samples collected from each MPS were used to measure protein and metabolite concentrations. Albumin secretion rates were used as an indicator of liver function (a). Barrier functions of gut (b) and lung (c) MPSs were assessed with TEER measurements. Endometrium MPS functionality was characterized with IGFBP-1 secretion rate to its apical medium (d).


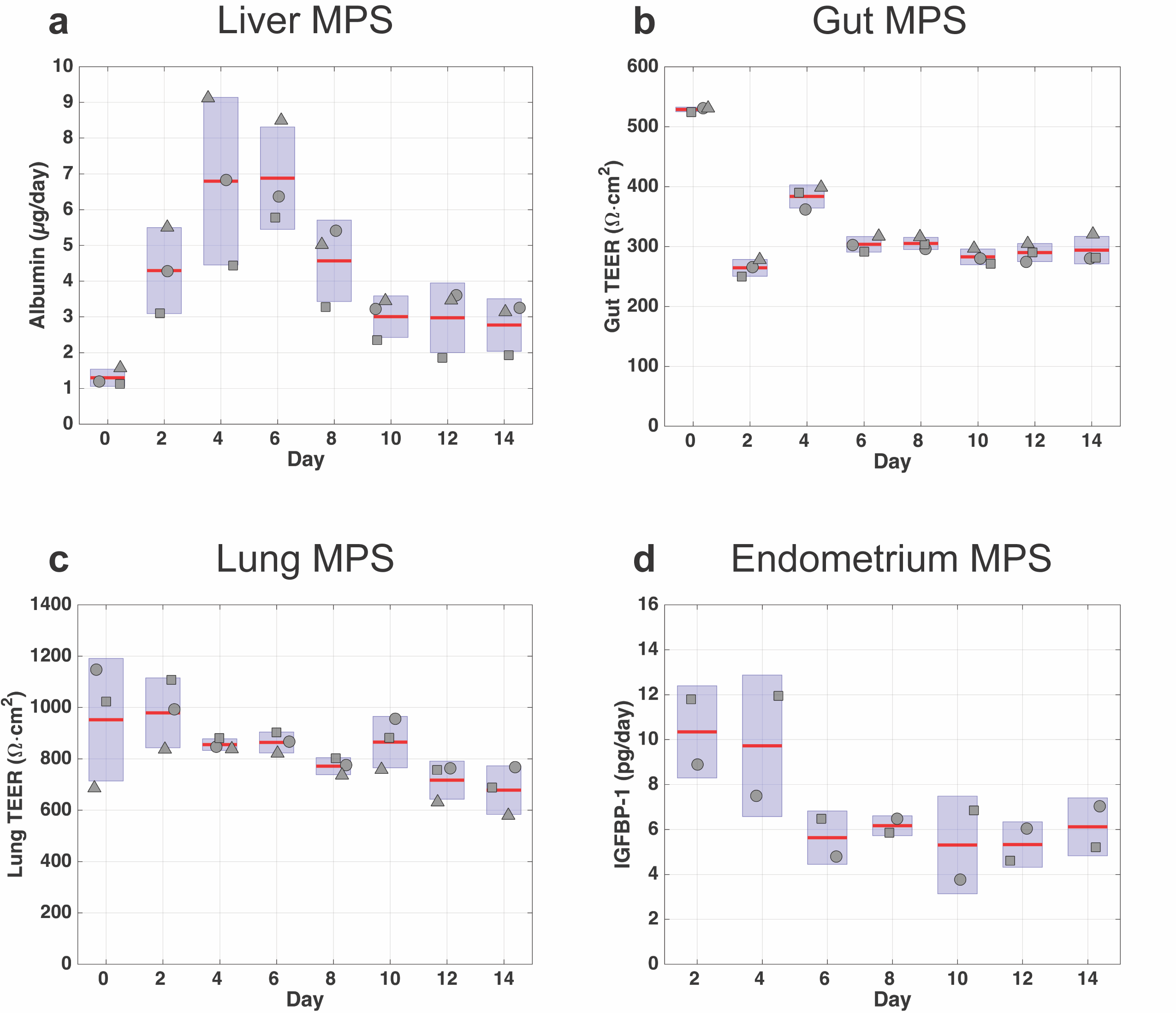


**Figure S5**.

Metrics of tissue function measured off platform (i.e., in isolation) for 3-week co-culture of 7 different MPSs. MPSs representing liver, gut, lung, endometrium, heart, pancreas, and brain were cultured in isolation. MPSs were grown according to the same methods as the maturation period described in methods, then culture was maintained in isolation rather than interaction. Samples collected from each MPS compartment were used to measure protein and metabolite concentrations. Albumin secretion rates were used as an indicator of liver function (a). Barrier functions of gut (b) and lung (c) MPSs were assessed with TEER measurements. Endometrium MPS functionality was characterized with IGFBP-1 secretion rate (d) to its apical medium. Heart MPS function was evaluated with beat frequency (e). C-peptide production rates (f) represented pancreas function. N-acetyl aspartate (NAA) concentrations (g) in the apical brain MPS indicated brain MPS functionality.


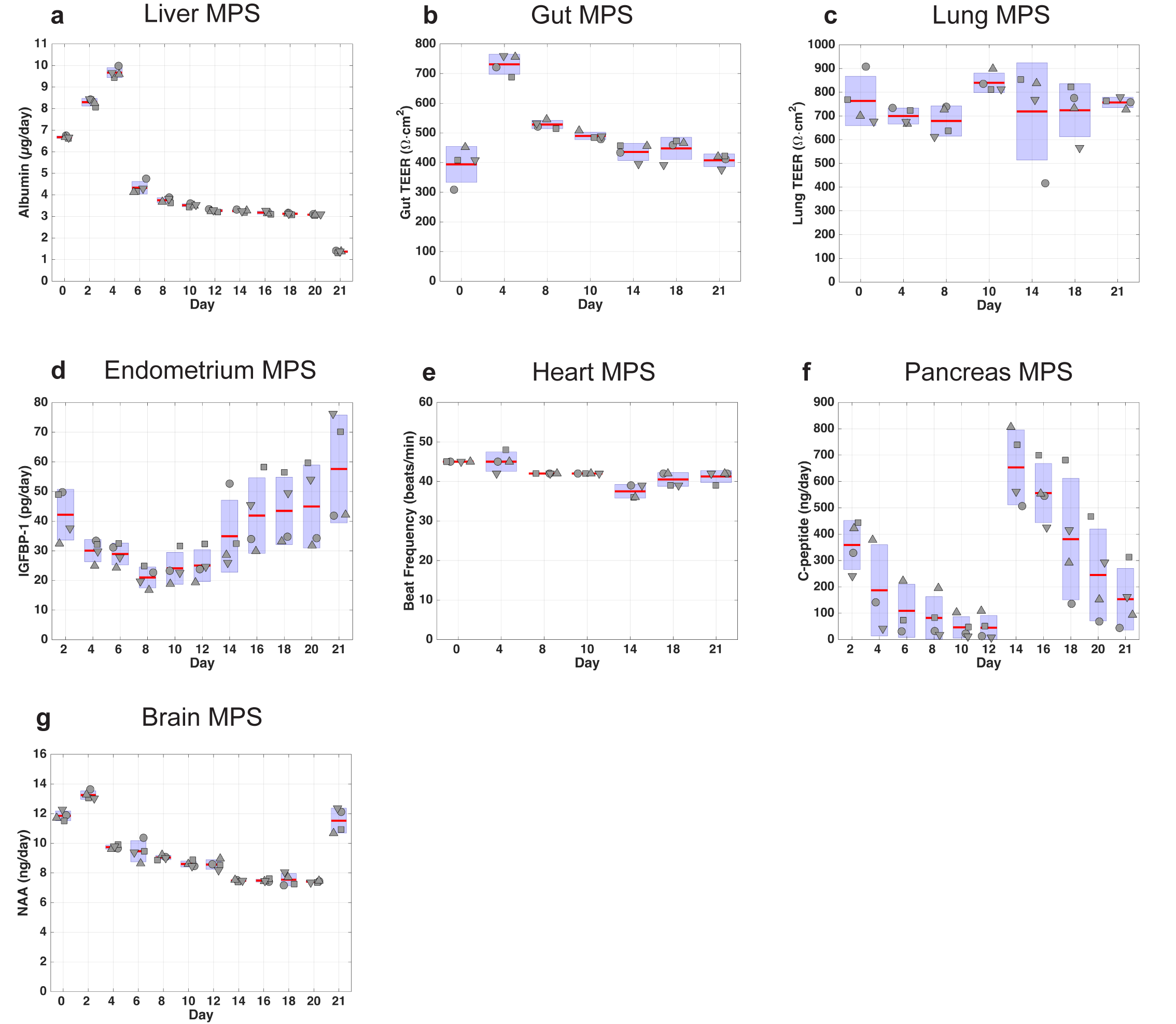


**Figure S6.**

Metrics of tissue function measured off platform (i.e., in isolation) for 4-week co-culture of 10 different MPSs. MPSs representing liver, gut, lung, endometrium, heart, pancreas, brain, skin, kidney, and skeletal muscle were cultured in isolation. MPSs were grown according to the same methods as the maturation period described in methods, then culture was maintained in isolation rather than interaction. Samples collected from each MPS compartment were used to measure protein and metabolite concentrations. Albumin secretion rates were used as an indicator of liver function (a). Barrier functions of gut (b) and lung (c) MPSs were assessed with TEER measurements. Endometrium MPS functionality was characterized with IGFBP-1 secretion rate (d) to its apical medium. Heart MPS function was evaluated with beat frequency (e). C-peptide production rates (f) represented pancreas function. N-acetyl aspartate (NAA) concentrations (g) in the apical brain MPS indicated brain MPS functionality. Barrier functions of skin (h) and kidney (i) MPSs were assessed with TEER measurements. Myostatin secretion was used an indicator of skeletal muscle function (j).


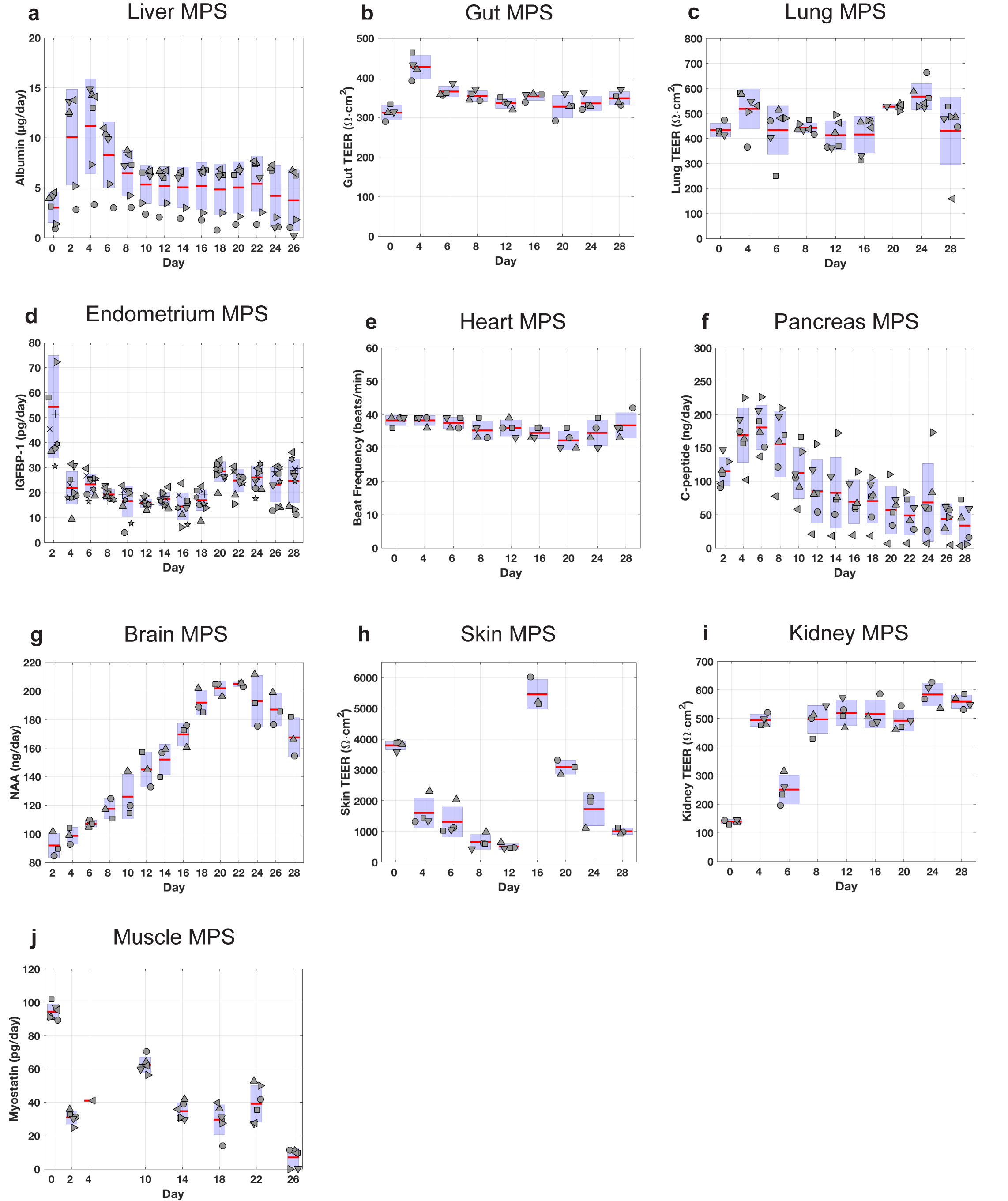


**N-acetyl aspartate analytical methods**

N-acetyl aspartate (NAA) was quantified by mass spectrometry as outlined below.

## Preparation of stock solutions: Standard Stock solutions @ 1.0 mg/mL in MeOH:Water 1:1.

Preparation of calibration standards working solutions: Cell Media

| Working Std Conc  (ng/mL) | Source Solution Conc.  (ng/mL) | Source Solution Vol (μL) | Solvent Volume  (μL) | Total Volume  (μL) | Solvent |
| --- | --- | --- | --- | --- | --- |
| 50,000 | 1,000,000 | 50 | 950 | 1000 | MEOH: water 1:1 |
| 5,000 | 50,000 | 100 | 900 | 1000 | MEOH: water 1:1 |
| 2,000 | 50,000 | 40 | 960 | 1000 | MEOH: water 1:1 |
| 1,000 | 2,000 | 500 | 500 | 1000 | MEOH: water 1:1 |
| 500 | 1,000 | 500 | 500 | 1000 | MEOH: water 1:1 |
| 200 | 500 | 400 | 600 | 1000 | MEOH: water 1:1 |
| 100 | 200 | 500 | 500 | 1000 | MEOH: water 1:1 |
| 50 | 100 | 500 | 500 | 1000 | MEOH: water 1:1 |
| 20 | 50 | 400 | 600 | 1000 | MEOH: water 1:1 |
| 5 | 20 | 250 | 750 | 1000 | MEOH: water 1:1 |

IS working solution: N-Acetylaspartic Acid-d3 @ 1,000 ng/mL in Acetonitrile:Water 10:90

Sample Matrix: Cell Media

Preparation of QC in MeOH: water 1:1: Cell Media

| QC Conc  (ng/mL) | Source Solution Conc.  (ng/mL) | Source Vol (μL) | Solvent Volume  (μL) | Total Volume  (μL) |
| --- | --- | --- | --- | --- |
| 20 | 50 | 100 | 900 | 1000 |
| 100 | 1000 | 100 | 900 | 1000 |
| 1000 | 2,000 | 500 | 500 | 1000 |

Preparation of STD and QC samples:

- Aliquot 25 µL of blanks, CTL-0, standard working solutions (5-5,000 ng/mL) and QCs and study samples into a 96-deep well plate.
- Aliquot 25 µL of cell media study samples.
- Add 25 µL IS working solution (NAA-d3 in ACN:Water 10:90 1,000 ng/mL) to all wells except for the blanks. For the blanks add 25 µL of water.
- Add 200 µL of 0.2% FA in water to all samples.
- Vortex at 1650 rpm for 3 minutes. Centrifuge at 3500 rpm for 5 minutes.

MS/MS #06 Parameters API 5500 ESI negative mode

| Compound | Parent m/z | | Product m/z | Scan (ms) | DP | CE | CXP |
| --- | --- | --- | --- | --- | --- | --- | --- |
| NAA | 173.9 | | 87.9 | 150 | -40 | -22 | -8 |
| NAA-d3 | 176.9 | | 90.9 | 150 | -40 | -22 | -8 |
| Other Detector Parameters: | | | | | | | |
| Ion Source TEM: | | 500 | GS1: 50 | GS2: 50 | IS: -4500 | NC: NA | EP:-10 |

*LC Method for Sample Analysis*

| LC gradient: | | HPLC System: | **Shimadzu Prominence** |
| --- | --- | --- | --- |
| Time (min) | %B | Column: | YMC Hydrosphere C18 |
| 0.20 | 5 | Flow rate: | 0.8 mL/min |
| 1.00 | 25 | Mobile phase A: | 10 mM Ammonium Acetate, 0.1% FA in Water |
| 2.10 | 95 | Mobile Phase B: | 0.1% FA in Acetonitrile |
| 2.20 | 95 | Injector Wash: | Methanol: water 1:1 |
|  | 5 | Injection Vol: | 10 μL |
| 3.00 | Stop |  |  |

# **Diclofenac, and 4-OH diclofenac analytical quantification methods**

The distribution and metabolism of diclofenac was investigated across three 7-way platforms (n=3). A diclofenac dose was administered in the apical side of the gut MPS (= 61.5 μM) and media samples (50 μL) from all the different MPS compartments were taken at pre-determined post-dose sampling times (at 24h and 48h from the mixing chamber and at 48h from all the other MPS compartments). All samples were analyzed for the presence of diclofenac (DCF) and its metabolite, 4-OH-diclofenac (4-OH-DCF).

The media samples were extracted by protein precipitation, and then were analyzed for Diclofenac, 4-hydroxy diclofenac and Hydrocortisone using LC-MS/MS methods with calibration curves.

Diclofenac, 4-hydroxy diclofenac and hydrocortisone were extracted from the media samples using protein precipitation. For the extraction of samples, protein precipitation of sample aliquots (20 µL) was initiated by adding 150 µL internal standard (a cocktail of Ritonavir and Warfarin at 10 ng/mL each in acetonitrile). After vortexing for 4 minutes, the samples were centrifuged at 3000 rpm for 15 minutes. A TomTec Quadra4 was used to transfer 100 µL of the resulting supernatant from each well into a clean 96-well plate containing 100 µL of water. The samples were then vortexed for 2 minutes followed by centrifugation for 5 minutes at 3000 rpm and then injected directly onto the LC-MS/MS for analysis.

The LC-MS/MS system consisted of a triple quadrupole mass spectrometer (API 4000) equipped with a Shimadzu Nexera UPLC system. The 3-in-1 LC-MS/MS method Diclofenac, 4-hydroxy diclofenac and hydrocortisone is described in the table below. Hydrocortisone results are not shown.

| General | |  | | | | | | | | | | | |
| --- | --- | --- | --- | --- | --- | --- | --- | --- | --- | --- | --- | --- | --- |
| Analyte | | Diclofenac, 4-hydroxy diclofenac and Hydrocortisone | | | | | | | | | | | |
| Matrix | | WEM:Brain basal media:Pancreas basal media:Heart basal media (75:10:5:10, v/v/v/v) | | | | | | | | | | | |
| Calibration Standard Concentrations | | 30/3/3, 100/10/10, 300/30/30, 1000/100/100, 3000/300/300, 5000/500/500, 10000/1000/1000 nM | | | | | | | | | | | |
| Internal Standard | | Cocktail of Ritonavir/Warfarin at 10/10 ng/mL in acetonitrile | | | | | | | | | | | |
| Sample volume | | 20 μL | | | | | | | | | | | |
| Extraction procedure summary | | The method utilized protein precipitation of the Diclofenac, 4-hydroxy diclofenac and Hydrocortisone and internal standard from media matrix using acetonitrile | | | | | | | | | | | |
| Chromatography Settings | |  | | | | | | | | | | | |
| Column type | | Acquity UPLC BEH C18, 1.7 μm, 50 mm × 2.1 mm, Waters | | | | | | | | | | | |
| Column switching | | 0.5-3.2 min to mass spec; all else to waste | | | | | | | | | | | |
| Column oven temperature | | 40°C | | | | | | | | | | | |
| Mobile phase composition | | A: Water:formic acid (100:0.1, v/v) | | | | | | | | | | | |
|  | | B: Acetonitrile:formic acid (100:0.1, v/v) | | | | | | | | | | | |
| Program | | Gradient | | | | | | | | | | | |
| Time (min) | | 0 | 2.5 | | 3.0 | | 3.1 | 3.8 | | | |  | |
| %B | | 20 | 100 | | 100 | | 20 | STOP | | | |  | |
| Autoinjector temperature | | 10°C | | | | | | | | | | | |
| Autoinjector wash solvent R0 | | Water:acetonitrile at 50:50 (v/v) | | | | | | | | | | | |
| Autoinjector wash solvent R3 | | Water:methanol:isopropyl alcohol:acetonitrile:DMSO at 20:20:20:20:20 (v/v/v/v) | | | | | | | | | | | |
| Flow rate | | ~600 μL/min | | | | | | | | | | | |
| Analysis time | | ~4.2 min | | | | | | | | | | | |
| Injection volume | | 4 μL | | | | | | | | | | | |
| Retention time | | Diclofenac = ~2.2 min | | | | | | | | | | | |
|  | | 4-hydroxy diclofenac = ~1.9 min | | | | | | | | | | | |
|  | | Hydrocortisone = ~1.4 min | | | | | | | | | | | |
|  | | Warfarin (IS) = ~2.0 min | | | | | | | | | | | |
| AB SCIEX Mass Spectrometry | | | | | |  | | | | |  | | |
| Parameter | | | | | | Value | | | | | Unit | | |
| Collision Gas (CAD) | | | | | | 6 | | | | | psig N2 | | |
| Curtain Gas (CUR) | | | | | | 30 | | | | | psig N2 | | |
| Ion Source Gas 1 (GS1) | | | | | | 60 | | | | | psig N2 | | |
| Ion Source Gas 2 (GS2) | | | | | | 60 | | | | | psig N2 | | |
| Ion Spray Voltage | | | | | | 5500 | | | | | V | | |
| Temperature (TEM) | | | | | | 600 | | | | | °C | | |
| Scan Duration | | | | | | 3.2 | | | | | min | | |
| Acquisition Method | | | | | |  | | | | |  | | |
| Compound | m/z transition | | | Ionization Mode | | Dwell Time (msec) | | | DP (V) | CE (V) | | | CXP (V) |
| Diclofenac | 295.921213.973 | | | ESP+ | | 50 | | | 41 | 80.1 | | | 16 |
| 4-hydroxy diclofenac | 312.060231.128 | | | ESP+ | | 50 | | | 45 | 29 | | | 19 |
| Hydrocortisone | 363.208121.000 | | | ESP+ | | 100 | | | 86 | 35 | | | 10 |
| Warfarin(IS) | 309.390163.000 | | | ESP+ | | 50 | | | 76 | 21 | | | 12 |

# **PBPK Models for endogenous albumin secretion and biodistribution to 4-MPS platform:**

Distribution of albumin was investigated in three 4-way platforms each consisting of a liver MPS (which produces albumin), a gut MPS, a lung MPS and an endometrium MPS (endo-MPS). A non-biological mixing chamber is connected to all MPSs, and flow partitioning from this chamber to every MPS is based on relative cardiac output in humans. See Fig. 2c in the main text for a flowchart of the 4-way platform. Cell culture media in the platform (i.e. for each MPS and the mixer) was completely changed at 2, 4 and 6 days after the start of the experiment. The systemic flow rate (*Q_sys_*) was stepwise increased from 5 ml/day on days 0-2 to 15 ml/day and 30 ml/day between 2-4 days and 4-6 days, respectively. Subsequently, albumin concentration in the media and actual media volumes in each MPS were determined.

The albumin distribution kinetics on the platform during the experiment were described mathematically by a set of ordinary differential equations as listed below. The media in each MPS is assumed to be homogeneous (well-mixed) based on prior experiments (not shown) evaluating mixing time of neutral density dyes. Distribution of albumin through the CaCo2-monolayer of the gut-MPS from the basal to apical side was neglected.

The production rate of albumin (*k_alb_*) was fitted to describe the distribution of albumin in the platform for different systemic flow rates. Fitting was performed by minimizing the sum of squared differences (least-square) between the albumin measurements and the model description for all MPSs and the mixing chamber, using all data points for all MPSs and all three platforms in each two-day period simultaneously. All other parameter values were specified based on operational values (for flowrates) and measured values (for MPS volumes). All parameter values, including the fitted values for *k_alb_* are provided in Table S2.

All simulations and parameter estimations were performed in Matlab (R2016a, The MathWorks, Inc., Natick, Massachusetts, United States).

**Mixing chamber concentrations (Eq.1):**


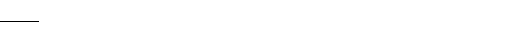


**Gut-MPS concentrations (Eq.2):**


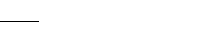


**Liver-MPS concentrations (Eq.3):**


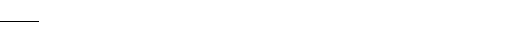


**Lung-MPS concentrations (Eq.4):**


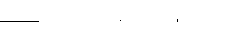


**Endo-MPS concentrations (Eq.5)**


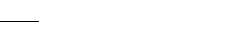


**Table S2. Parameter values of the 4-way platform model**

| **Parameter** | **Value** |
| --- | --- |
| Q_sys_ | 5 – 15 – 30 ml/day |
| Q_gut_ | 0.50*Q_sys_ |
| Q_liver_ | 0.30*Q_sys_ |
| Q_lung_ | 0.10*Q_sys_ |
| Q_endo_ | 0.10*Q_sys_ |
| V_mix_ | 2.0 ml ^(a)^ |
| V_gut_ | 1.2 ml ^(a)^ |
| V_liver_ | 1.2 ml ^(a)^ |
| V_lung_ | 0.72 ml ^(a)^ |
| V_endo_ | 0.84 ml ^(a)^ |
| k_alb, 0-2_ | 5.3 ± 0.7 1/day ^(b)^ |
| k_alb, 2-4_ | 7.7 ± 1.4 1/day ^(b)^ |
| k_alb, 4-6_ | 7.7 ± 0.4 1/day ^(b)^ |
|  |  |

(a) based on media recovery after 2, 4 and 6 days and averaged over n = 3 platforms.

(b) fitted to minimize the sum of squared differences between albumin measurements and model description, averaged over n = 3 platforms.

# **PBPK Models for exogenous drug administration to 7-MPS platform:**

A physiologically-based pharmacokinetic (PBPK) model was developed to describe and analyze the experimentally observed DCF and 4-OH-DCF concentrations in media across different compartments of the 7-MPS platform in an experiment where DCF was initially administered to the apical side of the gut MPS. The gut MPS was represented in the model as two separate compartments, referring respectively to the apical and the basolateral side of the cell culture, and the culture itself is permeable to DCF. For all the other MPS that were also in a transwell configuration (lung, heart, brain, endometrium) it was assumed that DCF / 4-OH-DCF distribution to the apical side of the transwell is negligible. DCF metabolism was assumed to take place only in the liver MPS. A fraction of DCF metabolism was assigned towards the measured 4-OH-DCF metabolite, while the remaining was assigned towards other non-measured metabolites. Based on previous experimental observations (43) a fraction of the parent drug is bound to the circulating media components (BSA) and thus only the unbound drug was assumed to be available for metabolism and diffusion across the gut epithelial barrier. Due to the fact that the formed micro-tissue volumes are very small relative to the surrounding media volumes, the model assumes that drug binding/partition into the tissues has a negligible effect on the media drug concentrations. The developed model can be mathematically described with a system of 18 ordinary differential equations, which are listed below.

**Lung MPS DCF concentrations (Eq.1):**

**Heart MPS DCF concentrations (Eq.2):**

**Brain MPS DCF concentrations (Eq.3):**

**Endometrium MPS DCF concentrations (Eq.4):**

**Gut MPS (apical side) DCF concentrations (Eq.5):**

**Gut MPS (basolateral side) DCF concentrations (Eq.6):**

**Pancreas MPS DCF concentrations (Eq.7):**

**Liver MPS DCF concentrations (Eq.8):**

**Mixing Chamber DCF concentrations (Eq.9):**

**Lung MPS 4-OH-DCF concentrations (Eq.10):**

**Heart MPS 4-OH-DCF concentrations (Eq.11):**

**Brain MPS 4-OH-DCF concentrations (Eq.12):**

**Endometrium MPS 4-OH-DCF concentrations (Eq.13):**

**Gut MPS (apical side) 4-OH-DCF concentrations (Eq.14):**

**Gut MPS (basolateral side) 4-OH-DCF concentrations (Eq.15):**

**Pancreas MPS 4-OH-DCF concentrations (Eq.16):**

**Liver MPS 4-OH-DCF concentrations (Eq.17):**

**Mixing Chamber 4-OH-DCF concentrations (Eq.18):**

where , and correspond respectively to volumes, flow rates and concentrations associated with the different model compartments. The subscripts *lu*, *he*, *br*, *en*, *gu*, *pa* and *li* refer to the lung, heart, brain, endometrium, gut, pancreas and liver MPS respectively, while the subscript *mi* refers to the mixing chamber. The apical and basal gut compartment are distinguished with the subscripts *gu(a)* and *gu(b)* respectively. Equations 1-9 refer to diclofenac (DCF), while equations 10-18 refer to the metabolite (4-OH-DCF). Cl_int(u)_ refers to the DCF unbound intrinsic clearance; fu_m_ is the fraction of drug unbound in the circulating medium; f_m_ is the fraction of DCF clearance that corresponds to the formation of the measured metabolite (4-OH-DCF); P and A are the permeability coefficient and the surface area respectively associated with the gut epithelial barrier. Any parameter (or concentration) referring to the metabolite is followed by a prime.

Subsequent to the DCF biodistribution experiment, the model was used to estimate the value of intrinsic clearance of unbound DCF, Cl_int(u)_, as well as f_m_, the fraction DCF being converted to the metabolite 4-OH-DCF, specific to the 7-way experiment environment and specific hepatocyte donor. To do so, all operational parameters were given the values set (flowrates) or measured (MPS volumes) in the experiment, gut permeability to DCF was used as measured in the isolated gut MPS experiments (data not shown), and the values of Cl_int(u)_ and f_m_ estimated by fitting the model to the experimental data, utilizing all measured data points simultaneously. The computational model parameters are shown in Table S3.

Prior to the DCF biodistribution experiment, the model was used to predict the DCF dose needed on the apical side of the MPS to result in a C_max_ in the 7-way platform mixer (representative of systemic circulation) similar to that observed clinically in patients (2-6 uM) (data not shown). These simulations used planned operating parameters (volumes, flowrates, gut area) and values of Cl_int(u)_ and f_m_ in the liver and P in the gut that had been measured from prior, isolated liver and gut MPS experiments, and simulated the media concentrations in all MPSs in the platform expected to result from a range of apical media concentrations. Based on these simulations a dose of 60 uM DCF on the apical gut was selected for the 7-way DCF biodistribution experiment.

**Table S3: Parameter values of the joint DCF / 4-OH-DCF model**

| **Parameter** | **Value** |
| --- | --- |
|  | 13.90 μL/min (SE = 1.17) ^(a)^ |
|  | 0.194 (SE = 0.009) ^(a)^ |
|  | 0.13 ^(b)^ |
|  | 17.8 x 10^-6^ cm/s ^(c)^ |
|  | 1.80 μL/min (26% of ) |
|  | 0.49 μL/min (7% of ) |
|  | 2.64 μL/min (38% of ) |
|  | 0.62 μL/min (9% of ) |
|  | 0.90 μL/min (13% of ) |
|  | 0.35 μL/min (5% of ) |
|  | 6.94 μL/min |
|  | 0.14 μL/min (2% of ) |
|  | 1.12 cm^2^ |
|  | 1.55 mL ^(d)^ |
|  | 1.19 mL ^(d)^ |
|  | 0.50 mL ^(d)^ |
|  | 0.91 mL ^(d)^ |
|  | 1.25 mL ^(d)^ |
|  | 1.84 mL ^(d)^ |
|  | 1.03 mL ^(d)^ |
|  | 3.27 mL ^(d)^ |
|  | 0.24 mL ^(d)^ |

^(a)^ Estimated parameters using the observed DCF and 4-OH-DCF data across the different platform compartments. Reported in parenthesis is the standard error associated with the parameter estimate. Parameter estimation was performed with NONMEM 7.3 (ICON Development Solutions, Ellicott City, Maryland, USA).

^(b)^ Experimentally determined for DCF in Tsamandouras et al (JPET, 2016). The same value has been also assumed with regard to the metabolite (4-OH-DCF).

^(c)^ Experimentally determined for diclofenac in the Gut MPS (unpublished in house data). The same value has been also assumed with regard to the metabolite (4-OH-DCF).

^(d)^ From media volume recovery measurements.

**MatLab script for cardiomyocyte beating frequency**

% CardioMyoQuant is a script that quantifies cardiomyocyte beating activity

% using a captured video segment

% Written by Timothy Kassis, last updated 12/30/15

clear all; clc;

%% Setup required variables

video_file = '96_Gelatin_Sample2-quicktime.mov';

pxl = 0.64; % 1 pixel = 0.64 um

vid_info = get(VideoReader(video_file));

duration = vid_info.Duration;

fs = vid_info.FrameRate;

dt = zeros(0,round(duration*fs));

av_mag = [];

%% Extract optical flow data from video file

% Open video file

videoReader = vision.VideoFileReader(video_file,'ImageColorSpace','Intensity','VideoOutputDataType','uint8');

% Convert image from uint8 to single

converter = vision.ImageDataTypeConverter;

% Create optical flow object for overlay

opticalFlow = vision.OpticalFlow('ReferenceFrameDelay', 1, 'OutputValue', 'Horizontal and vertical components in complex form');

% Create optical flow object for overlay

opticalFlow2 = vision.OpticalFlow('ReferenceFrameDelay', 1, 'OutputValue', 'Magnitude-squared');

% Creat object to overlay velocity lines

shapeInserter = vision.ShapeInserter('Shape','Lines','BorderColor','White');

% Create video player to play the video with the velocity overlay

videoPlayer = vision.VideoPlayer('Name','Motion Vector');

while ~isDone(videoReader)

frame = step(videoReader); % Open a new video frame

im = step(converter, frame); % Convert frame from unit8 to single

of = step(opticalFlow, im); % Estimate direction and speed

lines = videooptflowlines(of, 20);

if ~isempty(lines)

out = step(shapeInserter, im, lines);

step(videoPlayer, out);

end

mag = step(opticalFlow2, im); % Get magnitude

mag = double(mag);

av_mag = [av_mag mean(mean(nonzeros(mag)))]; % Calculate average magnitude for a frame

end

av_mag(1) = 0;

av_mag = av_mag*pxl; % Apply proper scaling

av_mag_smooth = smooth(av_mag,10);

% Close video players

release(videoPlayer);

release(videoReader);

%% Measure metrics including frequency and amplitude

magnorm = av_mag_smooth - mean(av_mag_smooth);

[pxx,f] = periodogram(magnorm,[],[],fs);

[pks, locs] = findpeaks(pxx,'NPeaks',1,'SortStr','descend');

%% Plot and display all results

figure();

% Plot smoothed signal

ax1 = subplot(3,1,1);

plot([0:(1/fs):(length(av_mag_smooth)-1)/fs]', av_mag_smooth);

ax1.XLim = [0 length(av_mag_smooth)/fs];

xlabel('Time (s)')

ylabel('Magnitude (\mum)')

title('Beating Signal - Time Domain')

% Plot frequency domain

ax2 = subplot(3,1,2);

plot(f,pxx,f(locs),pks,'or')

ax2.XLim = [0 2];

xlabel('Frequency (Hz)')

ylabel('Magnitude')

set((ax2),'XTick', 0:0.1:2)

title('Beating Signal - Frequency Domain')

text(f(locs)+0.04,pks,num2str(round(f(locs),3)),'HorizontalAlignment','left', 'FontSize',14);

% Plot histogram of magnitude

ax3 = subplot(3,1,3);

histogram(av_mag,200)

xlabel('Magnitude (\mum)')

ylabel('Occurance Frequency')

title('Histogram of Beating Amplitude')
